# The Al-Containing Silicates Modified with Organic Ligands and SnO_2_ Nanoparticles for Catalytic Baeyer-Villiger Oxidation and Aerobic Carboxylation of Carbonyl Compounds

**DOI:** 10.3390/nano13030433

**Published:** 2023-01-20

**Authors:** Jinyi Ma, Yong Wu, Qin Pan, Xiangdong Wang, Xiaoyong Li, Qiujuan Li, Xiaoshuai Xu, Yuan Yao, Yang Sun

**Affiliations:** 1 Department of Applied Chemistry, School of Chemistry, Xi’an Jiaotong University, No. 28, Xianning West Road, Xi’an 710049, China; 2 Xixian New District Xingyi Advanced Materials Technology Co., Ltd., Room 1046, 1st Floor, Hongdelou, Building No. 20, Science and Technology Innovation Port, Western China, Fengxi New City, Xixian New District, Xi’an 712000, China

**Keywords:** Baeyer-Villiger oxidation, aerobic carboxylation, Al-containing silicate, sol–gel, catalytic mechanism

## Abstract

The Baeyer-Villiger Oxidation (BVO) of ketones and aldehydes produce lactones and formates, while aerobic carboxylation of aldehydes manufactures carboxylic acids, both having high added value. This work prepared a series of Al-containing silicates modified with organic ligands and SnO_2_ nanoparticles, which were then employed as catalyst in BVO and carboxylation. Characterizations revealed the morphology of the synthesized catalyst was changed from micron-sized thin sheets to smaller blocks, and then to uniform nanoparticles (size of 50 nm) having the doped SnO_2_ nanoparticles with a size of 29 nm. All catalysts showed high BET surface areas featuring silt-like mesopores. In determining the priority of BVO and carboxylation, an influence evaluation of the parameters showed the order to be substrate > oxidant > solvent > catalyst. Cyclic aliphatic ketones were suitable for BVO, but linear aliphatic and aromatic aldehydes for carboxylation. Coordination of (*S*)-binaphthol or doping of Sn into catalyst showed little influence on BVO under *m*-CPBA, but the Sn-doped catalyst largely increased BVO under (NH_4_)_2_S_2_O_8_ and H_2_O_2_. Calculations revealed that the catalyst containing both Al and Sn could give BVO intermediates lower energies than the Sn-beta zeolite model. The present system exhibited merits including wider substrate scope, innocuous catalytic metal, greener oxidant, as well as lower catalyst cost.

## 1. Introduction

**Figure 1:**
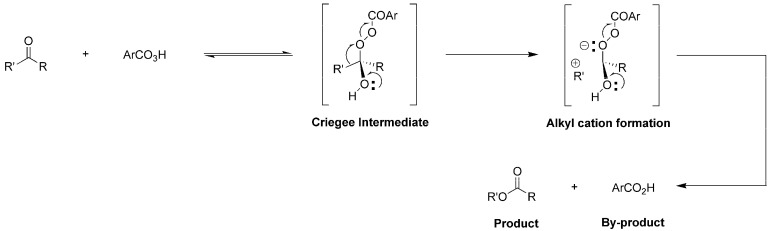
Proposed formation of Criegee intermediate and products.

The Baeyer-Villiger Oxidation (BVO) of carbonyl compounds such as ketones and aldehydes into lactones and formates, first developed by Baeyer and Villiger in 1899 [1], has shown great value in synthesis of sophisticated pharmaceuticals [2] and functional polymers [3], and stands as a model transformation in organic synthesis and industries [4]. Traditionally, most BVO reactions used peracid as terminal oxidant and followed a well-known mechanism. In detail, the carbonyl group of substrate (ketone or aldehyde) is nucleophilically attacked by peracid (oxygen source), producing a hemiperacetal (Criegee intermediate), which is further decomposed into one molecular ester (or lactone) and one molecular carboxylic acid (by-product) through simultaneous rearrangements of alkyl cation (R’^+^) and H^+^ (Figure 1) [5]. On the other hand, migration (rearrangement) of the two different substituents of one ketone (R and R’, Figure 1) gives different products, whose selectivity is denoted as “migratory aptitude” [6]. According to previous studies, the substituents thatare beneficial to stabilizing a positive charge migration (also to the stability of R’ cation) have a higher migratory aptitude [6]. This empirical rule has instructed the design of specific substrates for achieving target molecules.

Previously, BVO reactions were performed using stoichiometric or excessive peracids as oxidant including *m*-chloroperoxybenzoic acid (*m*-CPBA) [7] or trifluoroperoxyacetic acid [8]. Although satisfactory yields of BVO-type products were obtained, employment of peracids brought about two big problems, involving shock-sensitivity and appearance of equimolar carboxylic acids (unrecyclable by-product, Equation (1) [7]. Moreover, some peracids showed very low selectivity for BVO of substrates with unsaturated bonds (epoxidation prior to BVO), causing very poor BVO yields [6]. In addition, persulfate has also been used as terminal oxidant for BVO, but its full utilization needed the combinational use of catalyst having strong acidity, such as concentrated sulfuric acid (Caro’s acid) [9], inevitably leading to instrument corrosion and environmental pressure.

With the growing worldwide concerns for environmental protection, a greener oxidant and solvent as well as catalyst have been sought for catalytic BVO. For example, commercial H_2_O_2_ appeared to be a cleaner terminal oxidant for BVO, and transitional metal catalysts containing Pt [10] or Re [11] were put forward concomitantly, but these homogeneous catalysts often exhibited poor activity, and low chemoselectivity, mainly due to their low acidity. The high cost and non-recyclability of catalysts also retarded their wide application [10,11]. On the other hand, biocatalysts such as enzyme-based substances were explored for BVO of ketone in order to avoid metal pollution [12]. In practice, high chemoselectivity (migratory aptitude) could be obtained, but there were still problems with the availability, recovery and cost of the catalyst, along with product purification [12].

Furthermore, oxygen (O_2_) seemed to be another green and promising terminal oxidant for BVO reactions, but its main obstacle to wide application in BVO was its chemical inertness that cannot be fully activated by many acidic catalysts, such as *p*-toluenesulfonic acid (TfOH) [13] and metal catalyst [14]. Indeed, O_2_ was even more difficult to be activated in BVO than H_2_O_2_. Overall, the real large-scale application of greener oxidants such as H_2_O_2_ and O_2_ still lay in the combinational use of catalysts with great acidity.

Currently, using solid acid as BVO catalyst attracts wide and continuous interest, aiming to develop a highly efficient and chemoselective system for BVO using H_2_O_2_ or O_2_ as oxidant. These solid acids can be divided into several types according to their frameworks and corresponding properties. First, the periodic mesoporous organosilicas containing metalloporphyrin units were developed to catalyze BVO of cyclohexanone, whose high surface area and porosity facilitated conversion, but synthetic inconvenience of metalloporphyrin actually affected its large-scale application [15].

Secondly, metallic oxides or their mixtures also showed good activity for BVO. For example, the ordered mesoporous MgO featuring tunable pore diameter was prepared as alkaline catalyst, whose combination with H_2_O_2_ and benzonitrile catalyzed BVO of cyclohexanone smoothly [16]. Its only shortcoming was the use of benzonitrile as co-catalyst, yielding benzamide as by-product, affecting product purification [16]. Ma and co-workers synthesized a series of polyoxometalate catalysts featuring a two-dimensional crystalline porous structure for BVO of cyclohexanone, showing great conversion by using low-pressure O_2_ (1 atm) as oxidant and benzaldehyde as reductant [17]. This also revealed that mixing of multiple metal ions would create abundant empty d orbitals and crystal defects, subsequently activating the inert O_2_ oxidant [17].

Next, molecular sieves were explored and applied in various fields for many years, whose composition and mesoscopic structure can be designed directionally, giving many opportunities for catalytic BVO. In the early years, a series of Sn-zeolite beta catalysts were first established by Corma and co-workers in order to catalyze BVO reactions, and high conversions were obtained when H_2_O_2_ was used as oxidant [18]. Herein, Sn^4+^ was incorporated into the zeolite framework, exhibiting very high activity, and meanwhile Sn^4+^ would not catalyze H_2_O_2_-facilitated epoxidation as compared to Ti^4+^, Fe^3+^ or Cu^2+^, thereby avoiding un-wanted side products (epoxide) [18]. Nevertheless, these Sn-zeolite beta catalysts were synthesized through a hydrothermal process demanding corrosive F^−^ as template, and meanwhile weak basicity of F^−^ depressed nucleation, and subsequently lead to long-time crystallization [18].

In order to reduce the time of catalyst preparation, a steam-assisted conversion method was employed to synthesize Sn-beta zeolite rapidly through the formation of dry gel during the ageing procedure, and the resulting catalyst showed high selectivity to lactone, but this method still used F^−^ as template and provided only moderate conversion [19]. Later, F^−^-free synthesis of Sn-beta zeolite was put forward through dry gel method with satisfactory catalyst activity [20].

At the same time, there were continuous endeavors for promoting the activity of Sn-beta zeolite. In practice, the Sn content obtained using the traditional hydrothermal method was usually not high enough, because the radius of Sn^4+^ (0.71 Å) was too large to be incorporated into the silicate framework through ion substitution (0.41 Å for Si^4+^ radius) [21]. Therefore, a post-synthesis approach for implanting large transition metal ions such as Sn^4+^ into the zeolite framework appeared to be a powerful one. For example, the Sn^4+^ was incorporated into Sn-beta zeolite through the solid–gas reaction of highly siliceous Sn-beta zeolite with SnCl_4_ vapor [21]. Moreover, the post-synthetic removal of heteroatoms (Al or B) from the zeolite framework by acid washing could leave more defects or cavities to accommodate Sn^4+^ [22].

Lastly, in addition to the aforementioned solid acids, the potential offered by α-zirconium phosphate nanosheet [23], organic aerosol [24], as well as carbon nanotube [25] gradually came into sight, where Lewis and Brönsted acidities of the catalyst also showed a positive influence on catalytic BVO reactions, which greatly expanded the scope of BVO catalysts.

As for optimization of catalytic metal, contemporary studies have mainly focused on the design and application of transition metals such as Sn and Zr that were incorporated in highly silicious framework [1,22]. However, in practice, the effects of the aluminum catalyst on BVO were not completely revealed. Indeed, Al catalysts have had an important influence on various catalytic reactions. According to previous reports, an aluminum complex could catalyze Oppenauer (OPP) oxidation of alcohols, proving the Al^3+^ of homogeneous complex was an active Lewis acid center [26]. Furthermore, the immobilization of aluminum complexes into mesoporous supports would not decrease the catalytic activity and stability derived from their homogeneous counterpart, and meanwhile showed excellent recyclability in H-transfer reduction reactions of aldehydes and ketones [27]. The Cu oxide-modified alumina catalyst exhibited great activity for oxidative desulfurization of thiophene using H_2_O_2_ as oxidant, indicating that the interfaces of Cu-Al mixed oxide had Lewis acidity to activate molecules with lone pair electrons [28]. In addition, Al was usually much safer and more inexpensive than many other transition metals.

On the other hand, in view of the high added value of carboxylic acids found in pharmaceuticals, fine chemicals, and many other fields [29], the aerobic carboxylation (oxidation) of aldehydes into carboxylic acids aroused wide interests and have been catalyzed by many kinds of catalysts in the presence of versatile oxidants [29]. The previously used Mn or Cr oxide catalysts and stoichiometric oxidants such as sodium chlorite (NaClO_2_) also showed great efficiency for oxidation of carbonyl to carboxylic group [30]. Overall, catalytic conversion of aldehyde to carboxylic acid was not difficult to achieve in either laboratory or on an industrial scale, but the environmental risks coming from catalyst, solvent and oxidant, along with the production cost mainly relating to the catalyst, still deserved long-term attempts to improve upon it.

On the basis of the abovementioned previous results, this work aimed to develop a series of Al-containing silicate catalysts through a sol–gel process for BVO or aerobic oxidation of versatile carbonyl substrates, where high catalytic activity of Al, low catalyst cost, as well as greener oxidant and solvent were anticipated. Furthermore, taking into account that the homogeneous Al complex seemed highly active in oxidation, it was supposed that post-synthetic coordination of Al^3+^ on the catalyst surface with organic ligand (N- or O-containing) may further enhance the activity of Al.

Since Sn-beta zeolite showed satisfactory BVO activity instead of epoxidation, in addition to modification with the organic ligand, Sn was doped during a sol–gel process in order to create an Al-Sn-Si mixed oxide and facilitate BVO of carbonyl compounds. Furthermore, various oxidants including *m*-CPBA, (NH_4_)_2_S_2_O_8_ and H_2_O_2_ were loaded to study the synergy of the synthesized catalyst with its respective oxidant in BVO or other aerobic oxidations. Lastly, on the basis of theoretical calculations, a catalytic mechanism was summarized. In general, this work shows a new and interesting series of Al-containing silicate catalysts for BVO reactions, exhibiting likely potential in large-scale application.

## 2. Experimental

### 2.1. Starting Materials

The aluminum nitrate nonahydrate (Al(NO_3_)_3_·9H_2_O, 98%) and tin(II) chloride (SnCl_2_, anhydrous, 99%) were bought from Acros, and cetyltrimethylammonium bromide (CTAB, 99%), (*L*)-sodium lactate (99%) and tetraethyl orthosilicate (TEOS, 99%) were purchased from Alfa. The melamine (99%), (*S*)-(-)-binaphthol (98%), cyclohexanone (99%), cyclopentanone (99%), cyclohexanecarboxaldehyde (97%), butyraldehyde (98%) and benzaldehyde (99%) were bought from Sigma-Aldrich. The *m*-chloroperoxybenzoic acid (*m*-CPBA, 75%), ammonium persulfate ((NH_4_)_2_S_2_O_8_, 98%), hydrogen peroxide (H_2_O_2_, 30 wt.% water solution), along with ammonium hydroxide solution (NH_3_·H_2_O, 25 wt.% water solution) were bought commercially from Fluka. Other solvents were provided by local suppliers and not purified before use.

### 2.2. Instruments and Analytical Methods

The X-ray photoelectron spectroscopy (XPS) showed elemental composition and chemical state on the surface of the synthesized catalyst (depth of 0–3 nm), performed on Kratos Axis Ultra DLD with monochromatic Al Kα X-ray (1486.6 eV) as lighting source. By this means, the binding energy scale was calibrated by setting C 1s peak at 284.8 eV. The peaks obtained were further fitted by the Gaussian–Lorentz (G/L) product function with 30% Lorentzian. The wide-angle (2*θ* = 10°–80°) X-ray diffractions characterized the crystallinity of the synthesized catalyst, detected on PhilipsX’Pert Pro diffractometer by employing Cu-Kα radiation (λ = 1.5418 Å), whose interval was 0.05°s^−1^.

The porosity of the synthesized catalyst including BET surface area, pore volume, pore diameter, and pore size distribution illustrated differences in catalyst activity, which were measured on Micromeritics ASAP 2020 according to N_2_ adsorption isotherms at 77.35 K. The sample was first degassed at 150 °C in vacuum before detection. Its surface area was calculated using the multi-point Brunauer–Emmett–Teller (BET) method on the basis of adsorption data featuring relative pressure P/P_0_ of 0.06–0.3. Total pore volume was obtained from N_2_ that adsorbed at P/P_0_ = 0.97. Moreover, both pore volume and diameter were calculated by employing the Barrett–Joyner–Halenda (BJH) method.

The solvated size and zeta potential of the synthesized catalyst was tested on a Malvern Zetasizer Nano ZS90 spectrometer, characterizing the shape and stability of the sample in solvent. The acid amounts (involving both Lewis and Brönsted acidities) of the synthesized catalysts were determined employing *n*-butylamine titration with coeruleum bromocresol as indicator according to the literature method [31]. Thus, a quantity of the solid catalyst (300 mg) was mixed with *n*-butylamine (25.00 mL, 0.05 mol L^−1^ in toluene) in a conical flask of 250 mL. After shaking for 5 min under cover, 2-proponal (100 mL) a small amount of coeruleum bromocresol (one drop of diluted solution) was added. The solution obtained was then titrated by HCl solution (0.025 mol L^−1^ in H_2_O). When the solution color changed from blue to yellow, the end point of titration arrived, and the consumed volume of HCl solution was recorded. The amount of acid of the sample was viewed as the adsorbed amount of *n*-butylamine, obtained by subtraction from the total *n*-butylamine of the residues in solution that had been titrated by the HCl solution.

FT-IR spectroscopy characterized the functional groups of the synthesized catalyst, and was performed having dispersed the sample into a KBr pellet that was fixed on a Bruker Tensor 27, with wavenumber set to 400–4000 cm^−1^. Thermogravimetric analysis (TGA) gave information on the organic and inorganic composition of the synthesized catalyst, and was carried out on NETZSH TG 209C with TASC 414/4 controller under N_2_ protection, and heating rate 10 °C min^−1^ in the range 30–800°C. Differential scanning calorimetry (DSC) was measured on NETZSH DSC 214 with N_2_ protection, with heating rate of 10 °C min^−1^ in the range 30–300°C, characterizing the thermal effect of the synthesized catalyst’s phase change. The scanning electron microscopy (SEM) was performed on JEOL JSM-6700F at 20.0 kV in the absence of Au coating, while transmission electron microscopy (TEM) was carried out on JEOL JEM-200CX at 120 kV. The former method showed the morphology and size of the synthesized catalyst, while the latter exhibited its internal structure.

The conversion and respective product yield of BVO (also aerobic carboxylation) were determined on GCMS-QP2010 Plus, Shimadzu, using Rxi-5ms capillary column, of length 30 m, and internal diameter of 0.25 mm. For the GC part, column temperature was 60 °C, injection port temperature was 250 °C, sampling mode was split-flow, split-ratio was 26, and He was employed as carrier gas. For the MS part, ion source temperature was 200 °C, and interface temperature was 250 °C.

### 2.3. Synthesis of Al-Containing Silicate Catalysts Modified with Organic Ligands and SnO_2_ Nanoparticles

**Scheme 1:**
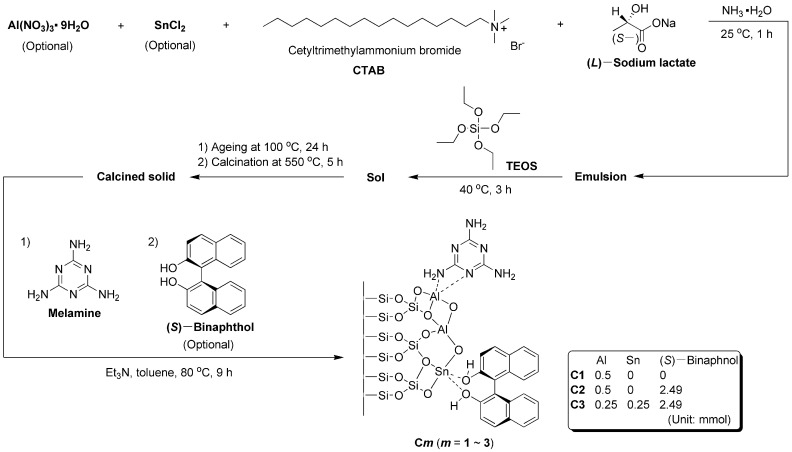
Synthesis of Al−containing silicate catalysts modified with organic ligands and SnO_2_ nanoparticles.

As shown in Scheme 1, Al(NO_3_)_3_·9H_2_O (C1-C2, 0.5 mmol; C3, 0.25 mmol), anhydrous SnCl_2_ (C1-C2, 0 mmol; C3, 0.25 mmol), CTAB (1.09 mmol), (*L*)-sodium lactate (0.43 mmol) were combined with NH_3_·H_2_O (100 mL) into a round-bottomed flask (250 mL). After stirring at 25 °C for 1 h, TEOS (9.02 mmol) was added, and the sol obtained was continuously stirred at 40 °C for 3 h. Then, the whole sol was transferred into an autoclave (150 mL), and the sealed autoclave was aged at 100 °C for 24 h. After being cooled to room temperature, the solids were collected by filtration under reduced pressure, and washed by distilled water (3 × 20 mL) and absolute ethanol (3 × 20 mL). The resulting solids were subsequently calcined at 550 °C for 5 h in muffle furnace and collected for future modification.

Next, the calcined solid (0.5 g), melamine (3.64 mmol) and triethylamine (20 mmol) were combined with dry toluene (100 mL) into a round-bottomed flask (250 mL). The resulting mixture was then refluxed at 80 °C for 6 h. The (*S*)–binaphthol (C1, 0 mmol; C2-C3, 2.49 mmol) was introduced, and the mixture obtained 10 was further refluxed at 80 °C for 3 h. After being cooled to room temperature, the solids were collected by filtration under reduced pressure, carefully washed by distilled water (3 × 20 mL) and absolute ethanol (3 × 20 mL), and then dried at 60 °C for 3 h in a baking oven, yielding C1-C3 respectively.

### 2.4. Catalytic BVO and Aerobic Carboxylation Reactions

The carbonyl compounds (2.0 mmol, covering cyclohexanone, cyclopentanone, cyclohexanecarboxaldehyde, butyraldehyde and benzaldehyde) were combined with solvent (10 mL, toluene, CH_2_Cl_2_, CH_3_CN and H_2_O, respectively) into a round-bottomed flask (100 mL) under magnetic stirring at room temperature. The oxidant (2.0 mmol, (NH_4_)_2_S_2_O_8_, *m*-CPBA and H_2_O_2_, respectively) and catalyst (2 mol% Al over substrate for C1-C2, and 2 mol% Al + Sn over substrate for C3, according to XPS data in Table 1) were added subsequently. The resulting mixture was further stirred at a pre-set temperature (80 °C or 40 °C) for 3 h. When the reaction was stopped, the mixture was filtered under reduced pressure, and the filtrate was detected on GC-MS for both identification and quantification.

### 2.5. Computational Methods

In this work, all calculations were performed with Gaussian 09 program [32]. The ONIOM (our own n-layered integrated molecular orbital and molecular mechanics) computational approach was used for geometry optimization and frequency calculations. The selected zeolite system was divided into two regions. The inner region, related with the reaction center, was taken as the high layer and conducted with the B3LYP hybrid density functional theory (DFT). The effective core pseudo-potential (ECP) LanL2DZ with the addition of single sets of diffuse function (α_p_ = 0.0174) and polarization function (α_d_ = 0.186), denoted as LanL2DZdp [33], was used for the Sn atom, while the 6–311++G(d,p) basis set was adopted for other atoms. The remaining extended zeolite framework was treated with the AM1 semi-empirical method. Vibrational frequency calculations were conducted to confirm each stationary point as a minimum or a transition state, and provided the thermal energy corrections. To obtain accurate energy evaluation, single-point energy calculations with the ωB97XD functional including the dispersion effect of van der Waals interactions were selected for the high-layered structure in the ONIOM scheme.

## 3. Results and Discussion

### 3.1. Elemental Composition and Chemical State of the Synthesized Catalyst

**Figure 2:**
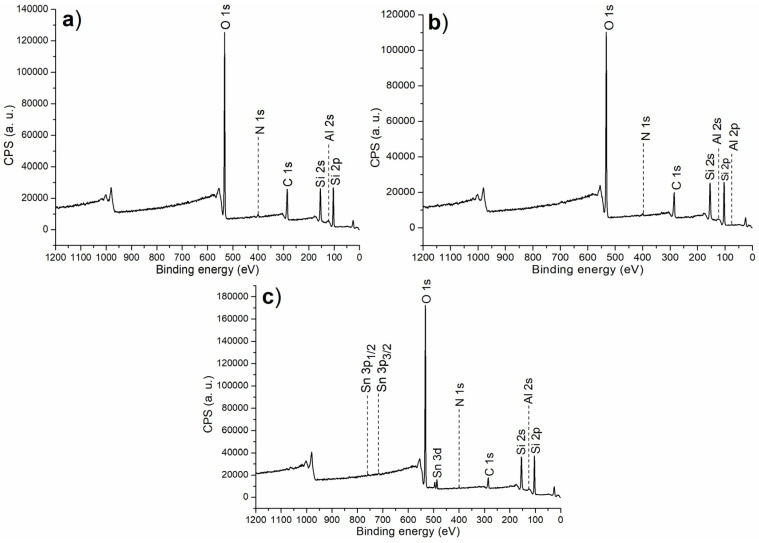
The XPS survey scan for the synthesized catalyst: (**a**) C1, (**b**) C2, (**c**) C3.

The XPS survey scan is summarized in Figure 2, with corresponding binding energy and atomic composition in Table 1. First of all, both C1 and C2 showed signals of N 1s photoelectrons (Table 1), indicating melamine was coordinated to Al centers on the surfaces of calcined solids (Scheme 1). Thus, when (*S*)–binaphthol was induced for synthesis of C2, the content of O was increased, but that of N was decreased (C2 vs. C1, Scheme 1 and Table 1), indicating that the originally coordinated melamine may be partially substituted with (*S*)–binaphthol during post-synthetic modification (Scheme 1).

If half of Al was replaced with Sn during the sol–gel process (C3 vs. C2, Scheme 1), both Al and Sn could be detected on the resulting catalyst (C3, Table 1; Figure 1), indicating the two metal ions can be incorporated through the present sol–gel. Furthermore, C3 showed higher content of O as well as lower content of N than C2 (Table 1), emphasizing Sn preferred to accommodate the O-containing ligand (binaphthol) than the N-containing one (melamine) (Scheme 1).

**Figure 3:**
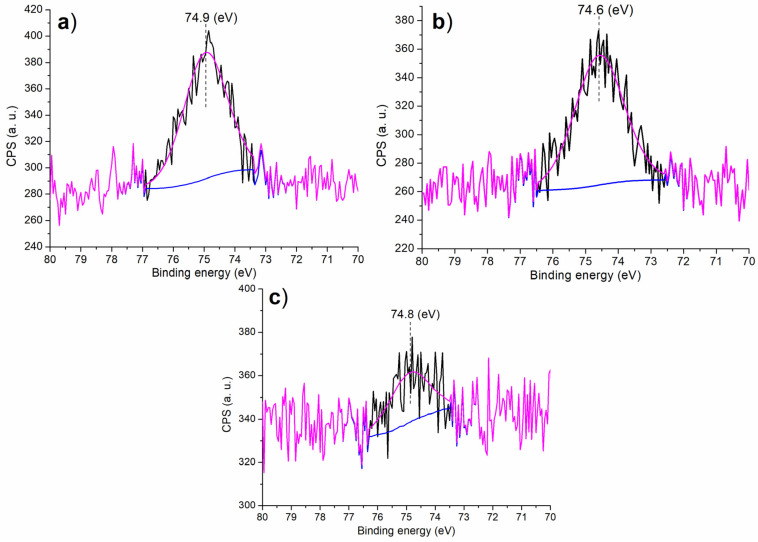
XPS measurement of Al 2p regions for the synthesized catalysts: (**a**) C1, (**b**) C2, (**c**) C3.

It is of further interest to discuss the chemical state of Al on the surface of the synthesized catalyst in order to understand the catalytic microenvironment. At first, there was only one component occurred at 74.9 eV on the Al 2p region of C1 (Figure 3a), characterizing the octahedrally-coordinated Al^3+^ that were fixed in the Al-Si mixed oxide framework coated with melamine (Scheme 1) [34]. This value was much higher than the 73.6 eV found in Al^3+^ of pure Al_2_O_3_ [34], mainly due to the mixing of Si^4+^ into Al_2_O_3_ phase that had much higher electronegativity. When (*S*)–binaphthol was introduced during post-synthetic modification (C2 vs. C1, Scheme 1), the N-containing ligand (melamine) was partially replaced with the O-containing one (binaphthol), causing a slightly decreased binding energy (Figure 3b vs. Figure 3a). However, when Sn^4+^ was doped in the sol–gel process (C3 vs. C2, Scheme 1), the resulting C3 showed higher binding energy of Al 2p photoelectron than C2 (Figure 3c vs. Figure 3b), probably indicating Sn^4+^ had higher electronegativity than Al^3+^.

**Figure 4:**
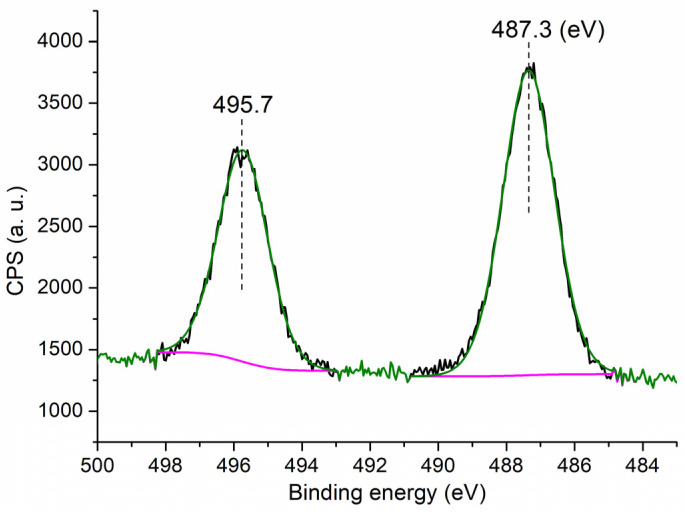
XPS measurement of the Sn 3d region for C3.

It is interesting to observe the chemical state of Sn on the surface of C3, which may show additional information on the catalytic component of C3. The C3 showed two peaks at 495.8 eV and 487.3 eV on the Sn 3d region (Figure 4), representing binding energies of Sn 3d_3/2_ and 3d_5/2_ photoelectrons, respectively, corresponding to the Sn^4+^ that is contained in the SnO_2_ phase [35]. In comparison with previously reported data, the binding energy of Sn 3d_5/2_ photoelectron of Sn^2+^ was found to be 485.8 eV, and that of metallic Sn was to be 485.3 eV [36]. Therefore, although divalent Sn (anhydrous SnCl_2_) was employed as the starting material, the Sn of C3 appeared to be tetravalent, probably due to oxidation during the sol–gel process (Scheme 1).

**Figure 5:**
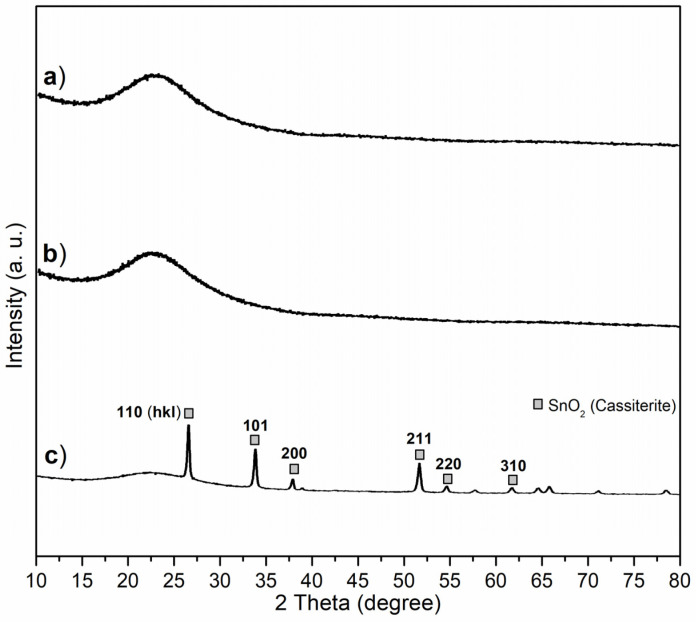
Wide-angle XRD patterns of powdered synthesized catalysts: (**a**) C1, (**b**) C2, (**c**) C3.

X-ray diffraction (XRD) was carried out to provide complementary information and illustrate the crystallinity and chemical state of the synthesized catalyst. Both C1 and C2 showed poor crystallinity, where the two broad bands (2*θ* = 15°–35°, centered at 23°, Figure 5a,b) reflecting the diffraction of the silicate backbone [34], and also indicating that Al^3+^ was highly dispersed into the silicate backbone without forming detectable Al_2_O_3_ or other Al-containing crystals. However, C3 showed diffractions of SnO_2_ (Cassiterite, PDF No. 41-1445) on wide-angle XRD (Figure 5c), proving tetravalence of Sn again.

In addition, there were three components on the C 1s region of C1, occurring at 285.3, 286.7 and 289.3 eV (Figure S1, Section S1, Supplementary Materials), which were indicative of C-O, C-N, and carboxyl group, respectively [37]. These carbon species may come from organic residues that formed during the sol–gel process, or from the organic ligand (melamine) attached in post-synthetic modification. The C2 showed three peaks that all shifted to lower binding energies compared to C1 (Figure S1b vs. Figure S1a), corresponding to saturated (sp^3^ hybridization) or unsaturated (sp^2^ hybridization) hydrocarbons, C-N bond, as well as carboxyl group [37], reflecting the effects of attached (*S*)–binaphthol (C2 vs. C1, Scheme 1). The binding energies of C 1s photoelectrons for C3 were quite close to those for C1 (Figure S1c vs. Figure S1a), indicating their similar average chemical states of C-containing components (C3 vs. C1, Scheme 1).

### 3.2. Textural and Other Physical Properties of Synthesized Catalyst

**Figure 6:**
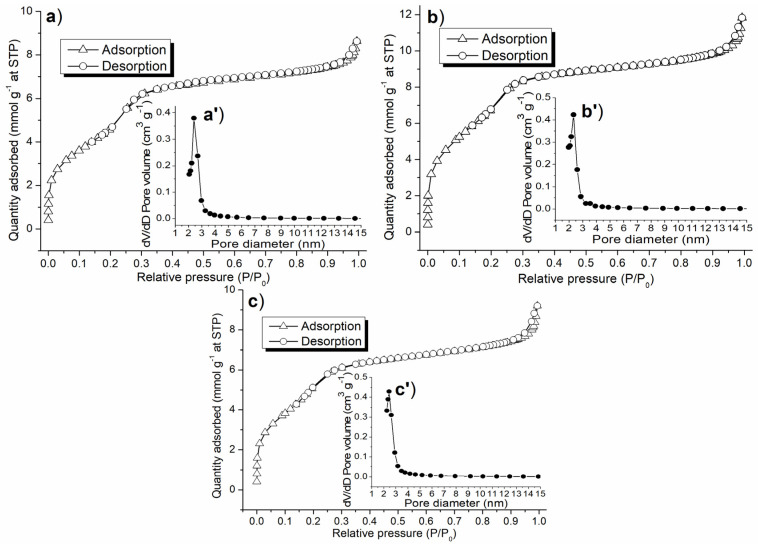
Nitrogen adsorption–desorption isotherm and pore size distribution of the synthesized catalyst: (**a**,**a′**) C1, (**b**,**b′**) C2, (**c**,**c′**) C3.

In light of the elemental and crystalline properties obtained so far, it was appropriate to detect textural properties of the synthesized catalyst in order to compare the synthesized catalysts. Above all, C1-C3 showed nitrogen physisorption isotherms with similar shapes, whose high-pressure sections (P/P_0_ = 0.3–1) conformed to a Type I isotherm, while low-pressure sections (P/P_0_ = 0–0.3) conformed to Type II (Figure 6a–c) [38], indicating they contained mesoporous structures [39]; this was also confirmed by their pore size distributions (Figure 6a’–c’). Since there were no hysteresis loops on these isotherms (Figure 6a–c), the mesopores had silt-like shapes [38].

As for the porosity, C1-C3 showed BET surface areas higher than 400 m^2^ g^−1^ (Table 2), making internal catalytic centers available to the substrate approaching. Furthermore, C2 showed a larger BET surface area, pore volume, pore radius, as well as lower density than C1 (Table 2), indicating (*S*)–binaphthol was attached to the silicate backbone, constructed new pores, and simultaneously decreased its bulk density. Lastly, C3 provided lower BET surface area and pore volume, along with higher bulk density than C2 (Table 2), indicating half substitution of Al with Sn actually gave a denser synthetic material (Scheme 1).

Accordingly, C2 showed a lower acid amount than C1, but its aqueous and solvated particle sizes (*d*_W_ and *d*_O_) and aqueous particle stability (*ζ*_W_) were promoted together (Table 3), meaning that the attachment of (*S*)–binaphthol occupied empty orbits of Al on the C2 surface, but increased stability of the solvated catalyst particle (Scheme 1). When half of Al was substituted with Sn during the sol–gel process, the resulting catalyst showed a higher acid amount, smaller aqueous and solvated particle sizes, as well as much better aqueous particle stability (*ζ*_W_) (C3 vs. C2, Scheme 1 and Table 3), indicating Sn may show higher acidity than Al in catalysis. Additionally, the size of SnO_2_ particle fixed on C3 was 29 nm (C3, Table 3), much smaller than the bulk crystallite size of C3 based on the BET surface area (C3, Table 2), indicating that the SnO_2_ particles were incorporated onto the Al-Si mixed oxide framework of C3.

### 3.3. Functional Group and Thermal Stability of Catalyst

As shown in Figure S2a (Section S2, Supplementary Materials), C1 showed a broad band centered at 3403 cm^−1^, indicating the stretching vibration of hydroxyl group on the catalyst surface. The following vibration occurred at 1635 cm^−1^ was which indicative of C=O stretching of an organic species [41], probably coming from (*L*)–sodium lactate that was introduced during the sol–gel process (Scheme 1). C1 further showed a series of vibrations at 1052, 965, 798 cm^−1^, which could be ascribed to Si-O stretching vibrations with different symmetry [42]. The peak that occurred at 439 cm^−1^ should be assigned to the Al-O vibration.

C2 showed a similar FT-IR spectrum to that of C1 (Figure S2b vs. Figure S2a), indicating they contained the same silicate framework (Scheme 1). The only difference was that C2 showed the stretching vibration of the Al-O bond at a wavenumber higher than the 439 cm^−1^ found on C1 (Figure S2b vs. Figure S2a), probably due to coordination of the (*S*)–binaphthol to the Al center of C2 (Scheme 1). Additionally, FT-IR of C3 seemed quite similar to C2 (Figure S2c vs. Figure S2b), proving the two synthesized catalysts had the same functional groups derived from both the silicate framework and attached ligands (Scheme 1). However, C3 showed the stretching vibration of metal (Al, Sn)-O bond at 439 cm^−1^, lower than that found on C2 (Figure S2c vs. Figure S2b), illustrating Sn-O stretching vibration had a lower energy than Al-O bond (Scheme 1).

**Figure 7:**
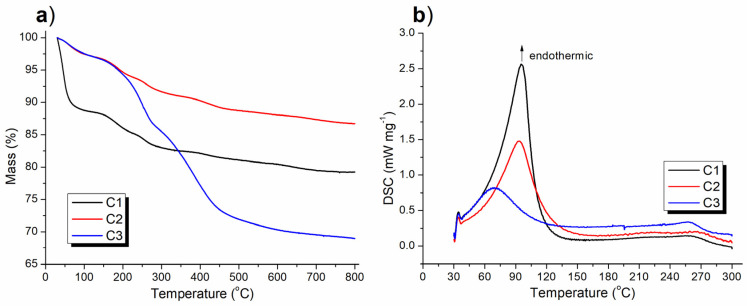
Thermal analysis of the synthesized catalyst: (**a**) TGA and (**b**) DSC curves.

TGA determined the content of organic components in the synthesized catalyst as well as its thermal stability, while DSC characterized the concomitant thermal effect. The C1 showed a weight loss of 11.64% at 30–135 °C (black line, Figure 7a), which was accompanied by a broad endothermic band which appeared at 50–135 °C (black line, Figure 7b), indicating the departure of adsorbed water as well as the attached ligand (melamine) under heating (C1, Scheme 1). The subsequent weight loss of 9.15% at 135–800 °C (black line, Figure 7a) represented removal of less volatile organic residues.

When (*S*)–binaphthol was introduced, the resulting catalyst (C2) showed a weight loss of 3.08% at 30–135°C (red line, Figure 7a), corresponding to a broad endothermic band which appeared at 50–135°C (red line, Figure 7b). Both of these tendencies were very similar to those exhibited by C1, although to a far or less extent (red vs. black, Figure 7a,b). Therefore, the weight loss of C1 at 30–135°C was caused by the departure of attached melamine under heating, while that of C2 at the same temperature range appeared to be the release of attached ligands including both melamine and (*S*)-binaphthol (Scheme 1). This clearly indicated that the introduction of (*S*)–binaphthol excluded some of the originally coordinated melamine during post-synthetic modification (Scheme 1). Furthermore, C2 showed TGA line drop in the range of 135–800°C in parallel to C1 (red vs. black, Figure 7a), proving the frameworks of C1 and C2 were basically identical, their difference coming from the post-synthetic modification (Scheme 1).

It was interesting to note that C3 and C2 had coincident TGA falling curves at 30–210°C (red and blue lines, respectively, in Figure 7a), which were both propelled by endothermic bands appearing at 50–135°C (red and blue lines respectively, Figure 7b), clearly indicating C3 and C2 contained the same amount and kind of attached ligands including melamine and (*S*)-binaphthol. However, due to the introduction of Sn during sol–gel synthesis of C3 (Scheme 1), C3 contained more less volatile organic residues than C2 (blue vs. red, Figure 7a).

### 3.4. Morphology and Internal Structure of Synthesized Catalyst

**Figure 8:**
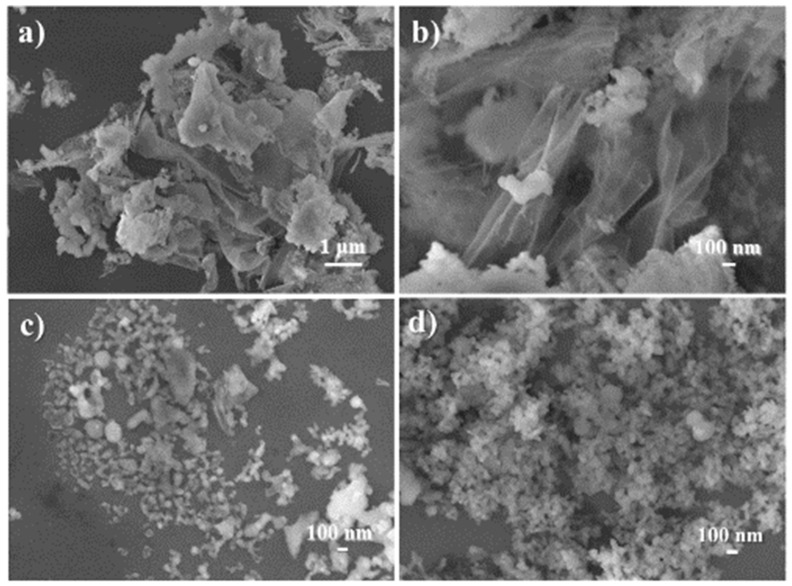
SEM images of synthesized catalysts:(**a**) C1 (magnification of 5000×), (**b**) C1 (30,000×), (**c**) C2 (30,000×), (**d**) C3 (30,000×).

**Figure 9:**
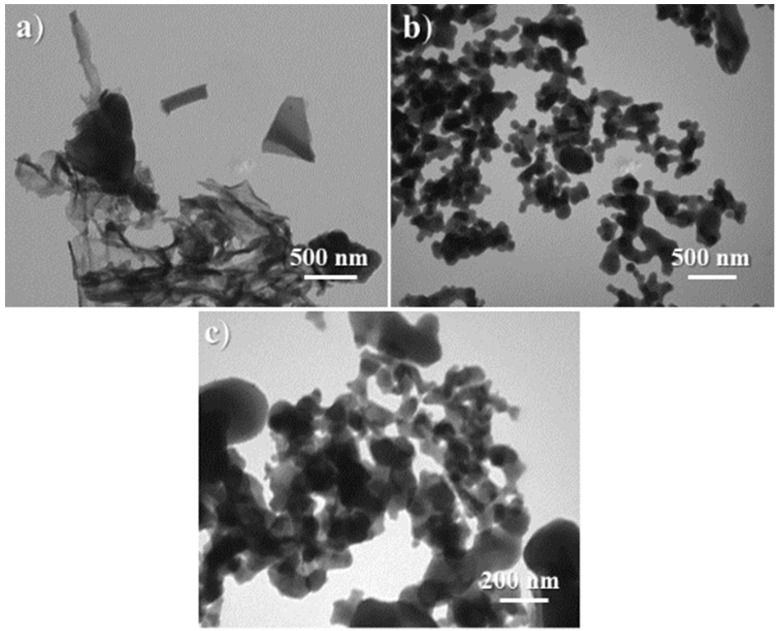
TEM images of the synthesized catalysts: (**a**) C1 (magnification of 50,000×), (**b**) C2 (50,000×), (**c**) C3 (100,000×).

Observation of the morphology and internal structure of the synthesized catalyst reflected the comprehensive effects of the aforementioned properties. At first, C1 was basically composed of many thin sheets more than 1 μm^2^ in size, along with a few blocks of 100–300 nm in size (Figure 8a,b and Figure 9a). Next, the loading of (*S*)–binaphthol in the sol–gel process scattered large thin sheets into smaller particles (Figure 8c vs. Figure 8a,b, and Figure 9b vs. Figure 9a), as expected from the coordination effect of binaphthol (C2 vs. C1, Scheme 1). Lastly, when half of Al was replaced with Sn (C3 vs. C2, Scheme 1), the obtained catalyst turned out to have uniform particle size of around 50 nm (Figure 8d and Figure 9c), and meanwhile the synthesized catalyst (C3) became much denser (Figure 8d vs. Figure 8c, Figure 9c vs. Figure 9b), which was also confirmed by bulk density (C3 vs. C2 in *ρ* item, Table 2).

### 3.5. Catalytic BVO and Aerobic Oxidation of Carbonyl Compounds

#### 3.5.1. Effect of Substrate

In all cases, the catalytic BVO and aerobic oxidation proceeded smoothly within 3 h, and it can be seen that the structure of the carbonyl substrate employed in this work played the dominant role in determining the outputs of the catalytic BVO or aerobic carboxylation. Firstly, when aliphatic ketones such as cyclohexanone and cyclopentanone were used as substrate, the BVO-type products (lactones) appeared to be the major product regardless of any other parameters including oxidant, solvent, catalyst or temperature (Table 4 and Table 5). Clearly, the present catalytic system employed a much cheaper metal (Al) to achieve high conversion for BVO transformation of aliphatic ketone to lactone.

Next, when cyclohexanecarbaldehyde was selected as substrate, both BVO (cyclohexyl formate) and aerobic carboxylation (cyclohexanecarboxylic acid) products were obtained (Table 6). However, transformation of other aldehydes such as *n*-butanal and benzaldehyde only produced carboxylic acid. This result probably indicated that the cyclohexyl cation was more stable than both propyl and phenyl cations during formation of Criegee intermediate, finally leading to BVO transformation (Scheme 1) [6]. In comparison, according to previous reports, most transformation of aldehyde under BVO conditions only yielded carboxylic acid, and only a few BVO-type examples came from conversion of benzaldehyde [43]. Therefore, this work showed a new and promising profile for BVO of cyclic aliphatic aldehyde.

#### 3.5.2. Effect of Terminal Oxidant

Apart from the substrate, the oxidant also showed an important influence on the outputs of catalytic BVO. When cyclohexanone, cyclopentanone and cyclohexanecarboxaldehyde were used as substrate, the effects of the oxidant on BVO outputs could be summarized in the order *m*-CPBA > (NH_4_)_2_S_2_O_8_ > H_2_O_2_ (Table 4, Table 5 and Table 6). On the one hand, it was previously reported that a peracid such as *m*-CPBA usually performed better than H_2_O_2_ for BVO [1,4], and the present catalytic system largely conformed this tendency (Table 4, Table 5 and Table 6). However, employing *m*-CPBA as the terminal oxidant invariably gave equimolar *m*-chlorobenzoic acid as by-product, and *m*-CPBA was also a shock-sensitive material [44], both of which features urge the exploration of greener oxidants such as O_2_.

Although average BVO performance of (NH_4_)_2_S_2_O_8_ was not as good as *m*-CPBA (entries 1–2 vs.3–5, Table 4; and 1 vs. 2–4, Table 6), the combinational use of (NH_4_)_2_S_2_O_8_ with C3 showed excellent conversion and chemoselectivity for BVO of cyclohexanone (entry 2, Table 4). Indeed, its conversion was much higher than that obtained using peroxomonosulfate (KHSO_5_)-supported acidic silica gel as catalyst in supercritical CO_2_, and chemoselectivity was also much better than that achieved by Caro’s reagent (K_2_S_2_O_8_ mixed with concentrated H_2_SO_4_) [45]. Therefore, both organic and inorganic oxidants could be well employed in the present system.

On the other hand, aerobic carboxylation of *n*-butanal could be carried out very well under all solvent, temperature and catalyst conditions (Table 7), proving *n*-butanal was active enough to accept both homogeneous and heterogeneous oxidants. However, when the substitute was changed to benzaldehyde, *m*-CPBA performed much better than H_2_O_2_, while (NH_4_)_2_S_2_O_8_ only worked in certain solvents (Table 8). This indicates that aerobic carboxylation using *m*-CPBA and H_2_O_2_ probably followed the mechanism derived from addition of the oxygen radical, but that using (NH_4_)_2_S_2_O_8_ may depend on oxidation of high valent sulfur [4,5].

#### 3.5.3. Effect of Solvent

In addition to analyzing substrate and oxidant, it was also significant to consider the effects of the solvent on catalytic BVO and aerobic carboxylation. Overall, organic solvents including toluene, CH_2_Cl_2_ and CH_3_CN had a good synergy with organic oxidants such as *m*-CPBA for BVO of cyclohexanone (entries 3–5, Table 4), cyclopentanone (entries 1–5, Table 5), cyclohexanecarbaldehyde (entries 2–4, Table 6), while the combination of organic solvent with inorganic oxidant usually provided much less outputs (entries 1 and 6, Table 4; entry 6, Table 5). However, the combination of inorganic solvent (H_2_O) with inorganic oxidant ((NH_4_)_2_S_2_O_8_) preferred aerobic carboxylation to BVO (entry 1, Table 6). Therefore, the miscibility between solvent and oxidant had a crucial influence on catalytic BVO outputs, and this influence exceeded the function of catalyst.

In catalytic aerobic carboxylation of benzaldehyde, the combinational use of organic solvent (CH_2_Cl_2_) with organic oxidant (*m*-CPBA) showed satisfactory conversion (entries 5–6, Table 8); the combination of water-soluble organic solvent (CH_3_CN) with H_2_O_2_ showed moderate outputs (entries 8–9, Table 8); while mixing of organic solvent (CH_2_Cl_2_, toluene) with inorganic oxidant (H_2_O_2_, (NH_4_)_2_S_2_O_8_) gave low to poor conversions (entries 1–2, 7, Table 8). Yet on the other hand, the mixing of inorganic solvent (H_2_O) with inorganic oxidant ((NH_4_)_2_S_2_O_8_) provided high conversion of aerobic carboxylation (entry 3, Table 8). It can be seen that the better miscibility between solvent and oxidant, the higher conversion of aerobic carboxylation obtained.

#### 3.5.4. Effect of Catalyst

Besides substrate, oxidant and solvent, the choice of catalyst also influenced the catalytic BVO outputs. When *m*-CPBA was used as terminal oxidant, C1 showed a slightly higher conversion than C2 in BVO of cyclopentanone (entries 4 vs. 5, Table 5), and C1 provided a little higher BVO yield (Product A) than C3 in BVO of cyclohexanecarbaldehyde (entries 2 vs. 3, Table 6). This indicated either the desirability of introducing (*S*)-binaphthol as the new coordinating ligand, or that doping of Sn showed little if any positive influence on catalyst activity in association with *m*-CPBA.

The substantial difference in catalyst activity came from the application of an inorganic oxidant. In practice, C1 turned out to be completely inactive for BVO of cyclohexanone, when either (NH_4_)_2_S_2_O_8_ or H_2_O_2_ was used as oxidant (entries 1 and 6, Table 4), which was in sharp contrast to the 100% conversion and complete chemoselectivity obtained by C3 under the same condition (entries 2 vs. 1, Table 4). This result indicated that the doping of Sn into Al-containing silicate gave the catalyst great miscibility with inorganic oxidants such as (NH_4_)_2_S_2_O_8_ and H_2_O_2_.

On the other hand, as for catalytic aerobic carboxylation, when *m*-CPBA was used as terminal oxidant, both C1 and C2 performed well in CH_2_Cl_2_, C2 seemed a little better than C1 (entries 6 vs. 5, Table 8). However, if *m*-CPBA was replaced with H_2_O_2_, C1 now looked better than C2 (entries 8 vs. 9, Table 8). Thus, introduction of (*S*)-binaphthol may promote catalyst miscibility with the organic oxidant (*m*-CPBA), whereas the lack of (*S*)-binaphthol modification results in a catalyst that performs better in association with inorganic oxidant (H_2_O_2_). From another viewpoint, C1 and C2 showed TOF values of 21.33 and 17.66 h^−1^, respectively, for oxidation of benzaldehyde into benzoic acid in CH_3_CN at 80 °C when H_2_O_2_ (30%) was used as terminal oxidant (entries 8–9, Table 8). These were comparable to the TOF of 1.60 h^−1^ derived from the biomimetic flavin (a quaternary ammonium salt)-catalyzed the same transformation in CH_3_CN at 85 °C with H_2_O_2_ (30%) as oxidant [46], indicating the promising application prospect of the present catalysts.

### 3.6. Theoretical Insights into Catalytic BVO Mechanism

The Sn-beta zeolite framework is viewed as an effective catalyst in the Baeyer-Villiger oxidation reaction, where H_2_O_2_ and cyclopentanone could be employed as oxidation and substrate respectively [47]. In this case, the hydroxyl open site was already confirmed more active than the closed site [48]. Therefore, the catalyzed reaction process at the open site of Sn-beta zeolite formed by the hydrolysis was explored. In this current work, C3 (Al-Sn mixed oxide) was also conjectured to be an effective catalyst in BVO of cyclopentanone (entry 6, Table 5), and was then simulated and the hypothesis tested accordingly.

**Figure 10:**
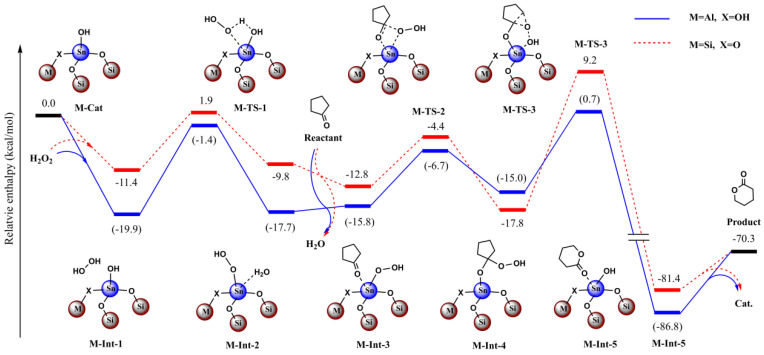
Calculated relative enthalpy (kcal/mol) profiles for the BVO of cyclopentanone under H_2_O_2_ catalyzed by C3 and Sn-beta zeolite model.

**Figure 11:**
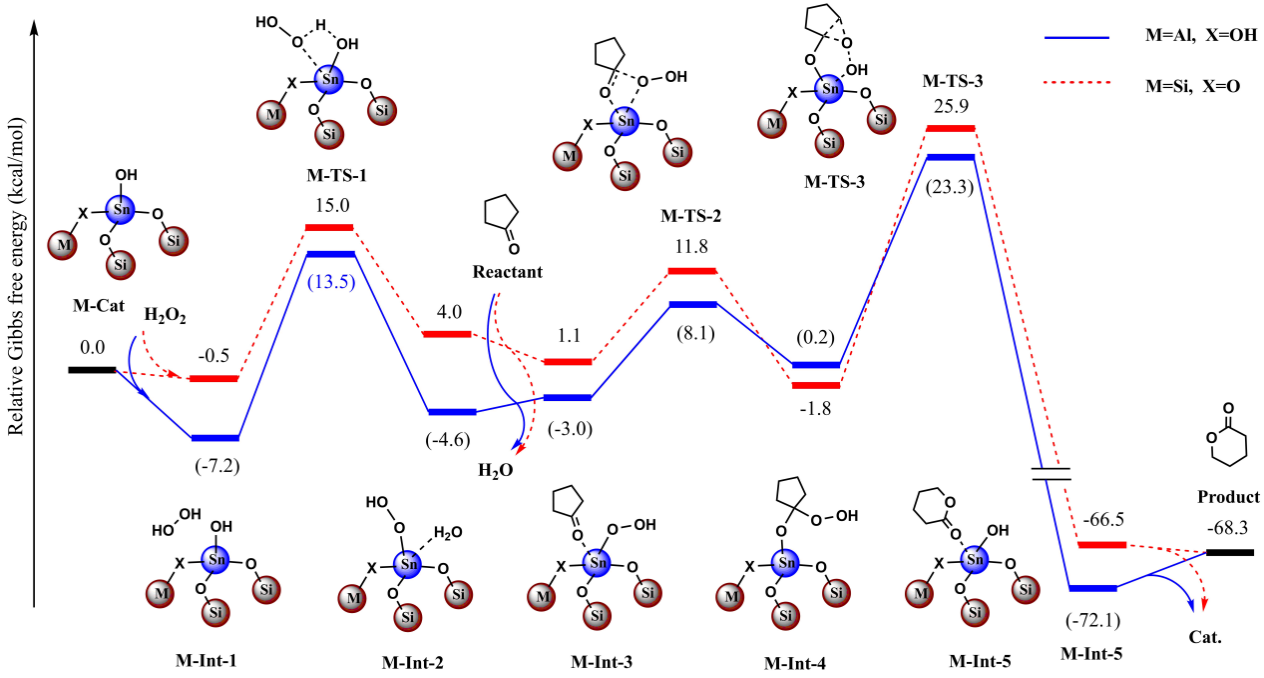
Calculated relative Gibbs free energies (kcal/mol) profiles for the BVO of cyclopentanone with H_2_O_2_ as terminal oxidant catalyzed by C3 and Sn-beta zeolite model.

We initially used the simple model (4T structure) which included the open-site Sn atom and four O atoms in the first coordination sphere (with SiH_3_ or H bound to them) to examine the possible reaction mechanism (Figure 10 and Figure 11, Figures S17 and S18, Section S8, Supplementary Materials). We considered both the stepwise and concerted mechanisms. In the stepwise mechanism, we found H_2_O_2_ would more favorably bind with the Sn site and form the Sn-OOH active intermediate via the dehydration (M-Int-2, Figure 9). The oxidation of cyclopentanone and recovery of the Sn-OH catalyst would then occur in the reaction (M-Int-3 to M-Int-5, Figure 9). However, it was found that the concerted oxidation of cyclopentanone without the formation of the Sn-OOH active intermediate would be unlikely due to the higher active energy barrier (not shown). In light of this, we only considered the favored pathway in the following two-layered ONIOM investigation (Figure 10 and Figure 11).

Moreover, due to lack of the constraining effect, we found the optimized structures deviated from the crystal structure to some extent in the simple model as stated above. To overcome the deviation and to balance the computational cost, we extended the catalyst model and adopted the ONIOM scheme. For the Sn-beta zeolite, the total model included the 45T structure and the central high-layered model including the 4T structure. In this way, the optimized structures were shown rationally, especially for the central high-layered model. To evaluate the effect of the Al site, we conducted the comparative computations with and without the effect of Al (Figure 10 and Figure 11).

If the Al site was far from the reactive center of Sn, we deemed that Al would have little effect on the catalyzed reaction and we did not take into account the Al in the calculations. For contrast, we then considered the case in which Al directly bound with the first-sphere O atom (Figure 10 and Figure 11). It was noted that the bridged O changed to OH to account for the neutral charge.

## 4. Conclusions

A series of Al-containing silicates modified with melamine, (*S*)–binaphthol and SnO_2_ nanoparticles were prepared through a sol–gel process. SEM and TEM revealed the synthesized Al-containing silicate modified with melamine was composed of thin sheets with a size of greater than 1 μm^2^; its further coordination with (*S*)–binaphthol dispersed the material into smaller blocks; while half substitution of Al with Sn in sol–gel yielded denser material containing uniform particles with an average size of 50 nm.

XRD revealed that Al^3+^ were highly dispersed into silicate frameworks of synthesized catalysts, but SnO_2_ particles of 29 nm in size were found on the Sn-doped sample. N_2_ physisorption revealed all synthesized catalysts had a BET surface area larger than 400 m^2^ g^−1^ along with silt-like mesopores. Other physical characterizations indicated the introduction of (*S*)–binaphthol as the additional ligand would decrease the acidity of the catalyst but increase catalyst stability in the solvent, but doping of Sn in the sol–gel process would increase acidity and solvated stability of synthesized catalyst.

The three synthesized catalysts were employed in BVO and aerobic carboxylation of carbonyl compounds. The extent of impact of the various parameters on BVO and carboxylation outputs could be summarized in the order substrate > oxidant > solvent > catalyst. At first, when cyclic aliphatic ketones were used as substrate, BVO-type products were dominant. When a cyclic aliphatic aldehyde was selected as substrate, aerobic carboxylation exceeded BVO. As for conversion of linear aliphatic and aromatic aldehydes, only carboxylation products were obtained.

Secondly, the effects of oxidant on BVO outputs could also be summarized in the order *m*-CPBA > (NH_4_)_2_S_2_O_8_ > H_2_O_2_. Next, the miscibility between solvent and oxidant had a crucial influence on catalytic BVO outputs, and the same tendency was found in catalytic aerobic carboxylation. Furthermore, introduction of (*S*)-binaphthol or doping of Sn showed little or slightly depressed influences on BVO activity of catalyst in association with *m*-CPBA, but doping of Sn in sol–gel synthesis of Al-containing silicate created a catalyst with great miscibility to inorganic oxidants such as (NH_4_)_2_S_2_O_8_ and H_2_O_2_. Lastly, simulation and calculation revealed that the doping of Sn into Al-containing silicate backbone will decrease the energies of consecutive BVO intermediates compared to those of the pure Sn-containing model (Sn-beta zeolite catalyst).

In general, this work put forward an interesting series of Al-containing silicate catalysts for BVO and aerobic carboxylation of versatile carbonyl compounds, showing potential for extending substrate scope, utilizing safer catalytic metal, employing greener terminal oxidant, as well as decreasing production cost, which are most relevant to future large-scale production.

## Figures and Tables

**Table 1:** Binding energy and atomic composition of element on catalyst surface.

Sample	C (1s)	O (1s)	N (1s)	Si (2p)	Al (2p)	Sn (3d)
C1	285.80 (25.69) ^a^	532.80 (43.63)	400.80 (0.78)	103.80 (29.48)	74.90 (0.43)	- ^b^
C2	284.80 (21.47)	532.80 (44.50)	398.80 (0.40)	102.80 (33.11)	74.50 (0.52)	-
C3	284.80 (11.06)	532.80 (52.74)	398.80 (0.11)	103.80 (35.70)	74.80 (0.15)	486.80 (0.24)

^a^ Binding energy (eV), along with atomic percentage (at%) in parentheses. ^b^ Not detected by instrument.

**Table 2:** Textural properties of synthesized catalysts.

Catalyst	*S*_BET_ (m^2^ g^−1^) ^a^	PV (cm^3^ g^−1^) ^b^	PR (nm) ^c^	*ρ* (g cm^−3^) ^d^	*d*_BET_ (nm) ^e^
C1	420	0.34	3.11	0.147	97
C2	588	0.40	3.16	0.138	73
C3	434	0.38	3.46	0.157	88

^a^ Surface area determined by BET method based on N_2_ adsorption. ^b^ BJH adsorption cumulative volume of pores having radii of 1.7 nm–300 nm. ^c^ Pore radius, BJH method on N_2_ adsorption. ^d^ Bulk density. ^e^ Crystallite size based on BET surface area: *d*_S_ = 6/(*S*_BET_·*ρ*), *ρ* bulk density [39].

**Table 3:** Other physical properties of the synthesized catalysts.

Catalyst	Acid Amount	*d*_XRD_	*d*_W_	*ζ*_W_	*d*_O_
	(mmol g^−1^) ^a^	(nm, SnO_2_) ^b^	(nm) ^c^	(mV) ^d^	(nm) ^e^
C1	1.12 × 10^−2^	-	1855	−152	292
C2	1.34 × 10^−3^	-	2866	−149	551
C3	4.30 × 10^−3^	29	1561	82.6	225

^a^ Determined by *n*-butylamine titration (Section 2.2). ^b^ Particle (SnO_2_) diameter originated from XRD based on Scherrer’s equation [40]: using 110 diffraction of SnO_2_ component for C3 (Figure 4c). ^c^ Aqueous particle size. ^d^ Zeta potential of particle in aqueous solution. ^e^ Size of particle in absolute ethanol.

**Table 4:** Catalytic BVO of cyclohexanone over synthesized catalyst.

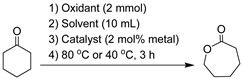						
Entry ^a^	Oxidant	Solvent	Temperature (°C)	Catalyst	Conversion (%) ^b^	Yield (%) ^c^
1	(NH_4_)_2_S_2_O_8_	toluene	80	C1	0	0
2		toluene	80	C3	100	100
3	*m*-CPBA	toluene	80	C1	62	62
4		CH_3_CN	40	C1	59	59
5		CH_2_Cl_2_	40	C2	54	54
6	H_2_O_2_ (30%)	CH_3_CN	80	C1	0	0

^a^ Experimental details as shown in Section 2.4; cyclohexanone, 2 mmol. ^b^ Conversion of cyclohexanone (substrate) to all possible products, determined by GC-MS (Figures S3–S7, Section S3, Supplementary Materials). ^c^ Yield of oxepan-2-one based on substrate, determined by GC-MS (Section S3, Supplementary Materials).

**Table 5:** Catalytic BVO of cyclopentanone over synthesized catalyst.

Entry ^a^	Oxidant	Solvent	Temperature (°C)	Catalyst	Conversion (%) ^b^	Yield (%) ^c^
1	*m*-CPBA	CH_3_CN	80	C3	44	44
2		toluene	80	C1	100	100
3		toluene	40	C2	100	100
4		CH_2_Cl_2_	40	C1	89	89
5		CH_2_Cl_2_	40	C2	86	86
6	H_2_O_2_ (30%)	CH_3_CN	80	C3	16	16

^a^ Experimental details as shown in Section 2.4; cyclopentanone, 2 mmol. ^b^ Conversion of cyclopentanone (substrate) to all possible products, determined by GC-MS (Figures S8–S10, Section S4, Supplementary Materials). ^c^ Yield of tetrahydropyran-2-one based on substrate, determined by GC-MS (Section S4, Supplementary Materials).

**Table 6:** Catalytic BVO and aerobic carboxylation of cyclohexanecarbaldehyde over synthesized catalyst.

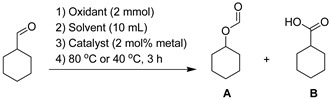						
Entry ^a^	Oxidant	Solvent	Temperature (°C)	Catalyst	Conversion (%) ^b^	Yield (%) ^c^
1	(NH_4_)_2_S_2_O_8_	H_2_O	80	C3	79	79(B)
2	*m*-CPBA	toluene	80	C1	90	27(A),63(B)
3		toluene	80	C3	93	25(A),68(B)
4		CH_2_Cl_2_	40	C3	69	35(A),34(B)

^a^ Experimental details as shown in Section 2.4; cyclohexanecarbaldehyde, 2 mmol. ^b^ Conversion of cyclohexanecarbaldehyde (substrate) to all possible products, determined by GC-MS (Figures S11 and S12, Section S5, Supplementary Materials). ^c^ Yield of each product based on substrate, determined by GC-MS (Section S5, Supplementary Materials).

**Table 7:** Catalytic aerobic carboxylation of *n*-butanal over synthesized catalyst.

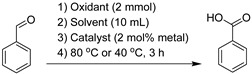						
Entry ^a^	Oxidant	Solvent	Temperature (°C)	Catalyst	Conversion (%) ^b^	Yield (%) ^c^
1	(NH_4_)_2_S_2_O_8_	toluene	80	C2	100	100
2	*m*-CPBA	toluene	80	C1	100	100
3		toluene	80	C2	100	100
4		CH_2_Cl_2_	40	C3	100	100
5		CH_2_Cl_2_	40	C1	100	100
6		CH_2_Cl_2_	40	C2	100	100
7		CH_3_CN	40	C1	100	100
8		CH_3_CN	40	C2	100	100
9	H_2_O_2_ (30%)	CH_3_CN	80	C1	100	100

^a^ Experimental details as shown in Section 2.4; *n*-butanal, 2 mmol. ^b^ Conversion of *n*-butanal (substrate) to all possible products, determined by GC-MS (Figures S13 and S14, Section S6, Supplementary Materials). ^c^ Yield of butyric acid based on substrate, determined by GC-MS (Section S6, Supplementary Materials).

**Table 8:** Catalytic aerobic carboxylation of benzaldehyde over synthesized catalyst.

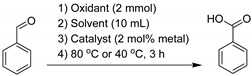							
Entry ^a^	Oxidant	Solvent	Temperature (°C)	Catalyst	Conversion (%) ^b^	Yield (%) ^c^	TOF(h^−1^) ^d^
1	(NH_4_)_2_S_2_O_8_	toluene	80	C1	0	0	0
2		toluene	80	C2	0	0	0
3		H_2_O	80	C3	70	70	23.33
4		CH_2_Cl_2_	40	C1	61	61	20.33
5	*m*-CPBA	CH_2_Cl_2_	40	C1	96 ^e^	79	26.33
6		CH_2_Cl_2_	40	C2	99 ^e^	82	27.33
7	H_2_O_2_ (30%)	CH_2_Cl_2_	40	C3	50	50	16.66
8		CH_3_CN	80	C1	64	64	21.33
9		CH_3_CN	80	C2	53	53	17.66

^a^ Experimental details as shown in Section 2.4.; benzaldehyde, 2 mmol. ^b^ Conversion of benzaldehyde (substrate) to all possible products, determined by GC-MS (Figures S15 and S16, Section S7, Supplementary Materials). ^c^ Yield of benzoic acid based on the substrate, determined by GC-MS (Section S7, Supplementary Materials). ^d^ Turnover frequency, mol_dominantproduct_ mol_metalofcatalyst_^−1^ (time, h)^−1^. ^e^ In addition to benzoic acid, phenethyl ether appeared as by-product, formed during an unknown catalytic process (Section S7, Supplementary Materials).

## Data Availability

We all authors would like to share our research data.

## Supplementary Materials

The following supporting information can be downloaded at: https://www.mdpi.com/article/10.3390/nano13030433/s1, Section S1: XPS measurement of C 1s region for the synthesized catalyst (Figure S1); Section S2: FT-IR spectra of the synthesized catalyst; Section S3: GC-MS examples for Table S4; Section S4: GC-MS examples for Table S5; Section S5: GC-MS examples for Table S6; Section S6: GC-MS examples for Table S7; Section S7: GC-MS examples for Table 8; Section S8: Optimized structures (distance in Å) for the oxidation of cyclopentanone under H_2_O_2_ catalyzed by C3 and Sn-beta zeolite model.
