# Supplementary material for: The Al-Containing Silicates Modified with Organic Ligands and SnO2 Nanoparticles for Catalytic Baeyer-Villiger Oxidation and Aerobic Carboxylation of Carbonyl Compounds
Source: Nanomaterials (Basel). 2023 Jan 20;13(3):433. doi: 10.3390/nano13030433 (PMC9919301; doi:10.3390/nano13030433)
Supplement: Supplementary file 1 [file nanomaterials-13-00433-s001.zip › nanomaterials-2145506-supplementary.pdf]

nanomaterials

Supplementary Materials

The Al-Containing Silicates Modified with Organic Ligands and SnO_2 Nanoparticles for Catalytic Baeyer-Villiger Oxidation and Aerobic Carboxylation of Carbonyl Compounds

Jinyi Ma[1], Yong Wu [1,2], Qin Pan [1,2], Xiangdong Wang [1,2], Xiaoyong Li [1,2], Qiujuan Li[1,2], Xiaoshuai Xu[1,2], Yuan Yao [1,2] and Yang Sun [1,2,*]

[1] Department of Applied Chemistry, School of Chemistry, Xi'an Jiaotong University, No. 28, Xianning West Road, Xi'an 710049, China
[2] Xixian New District Xingyi Advanced Materials Technology Co., Ltd., Room 1046, 1st Floor, Hongdelou, Building No. 20, Science and Technology Innovation Port, Western China, Fengxi New City, Xixian New District, Xi'an 712000, China
* Correspondence: sunyang79@mail.xjtu.edu.cn; Tel.: +86-29-8266-3914; Fax: +86-29-8266-8559

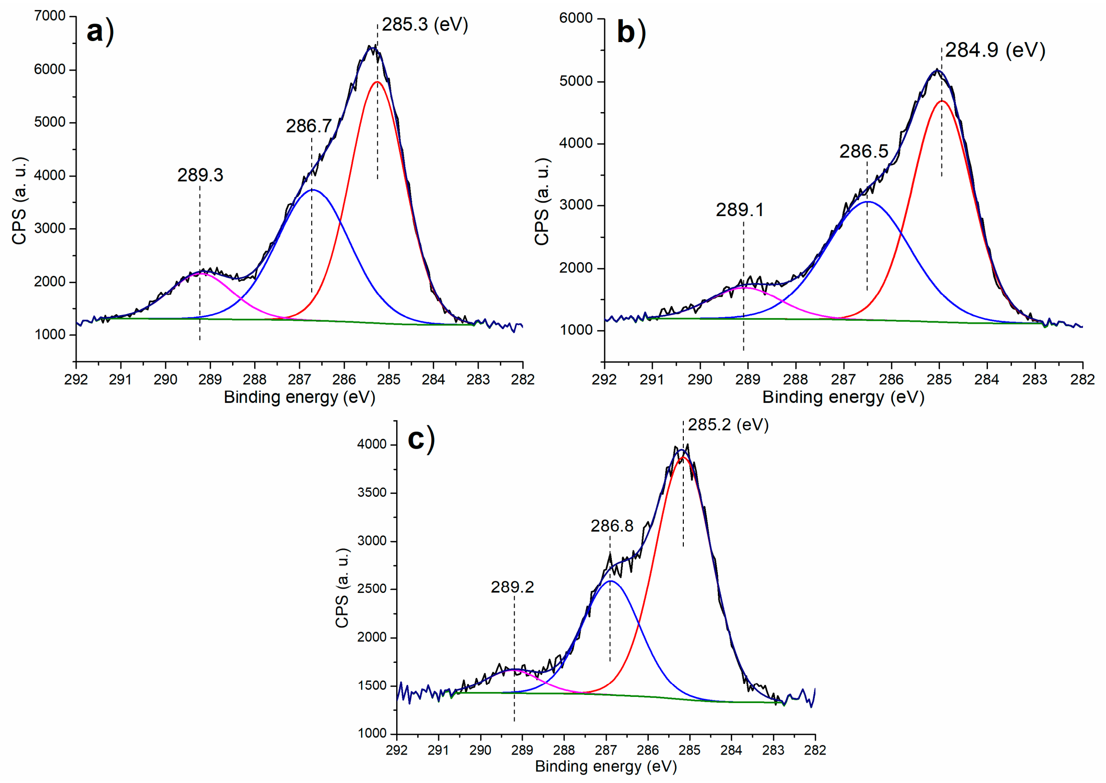

Figure S1. XPS measurement of C 1s region for the synthesized catalyst: (**a**) C1, (**b**) C2, (**c**) C3.

S2. FT-IR spectra of the synthesized catalyst

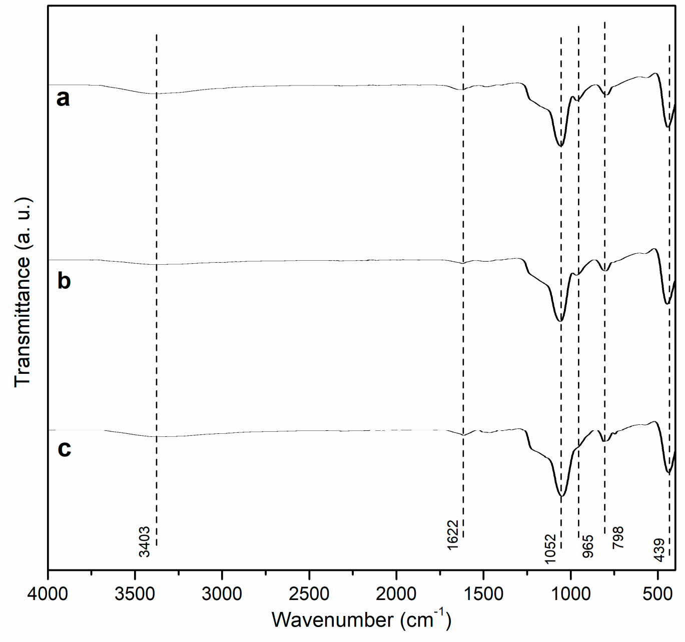

Figure S2. FT-IR spectra of the synthesized catalyst: (**a**) C1, (**b**) C2, (**c**) C3.

S3. GC-MS examples for Table 4

Entry 1, Table 4

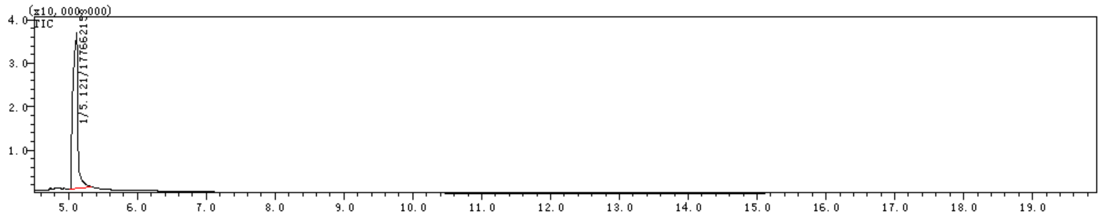

Figure S3. GC part of GC-MS for Entry 1, Table 4.

1) The peak for t_R = 5.121 min is:

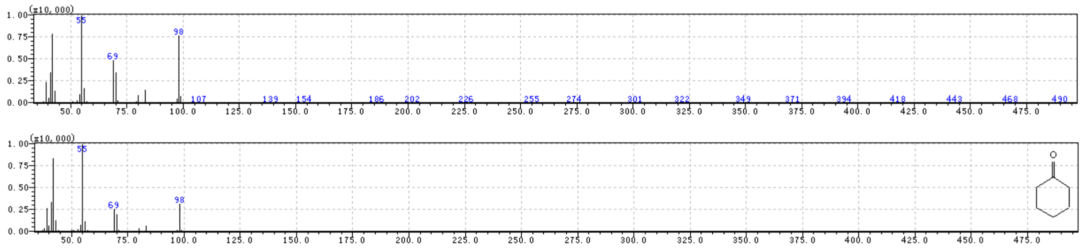

GC-MS: calcd. for $C_6H_{10}O$ 98, found 98 ($C_6H_{10}O$). Table 4.

2) Entry 2, Table 4

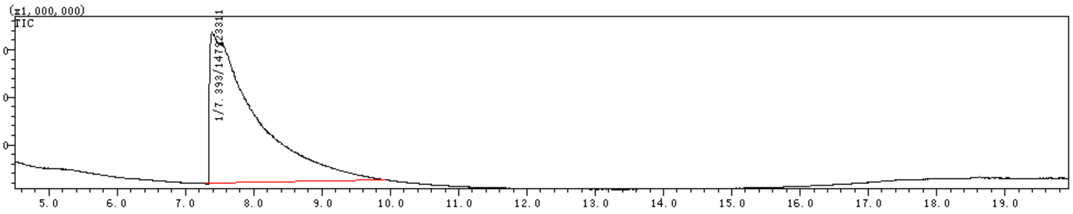

Figure S4. GC part of GC-MS for Entry 2, Table 4.

In this image, data format is peak number / retention time (min) / integral area.

The peak for t_R = 7.393 min is:

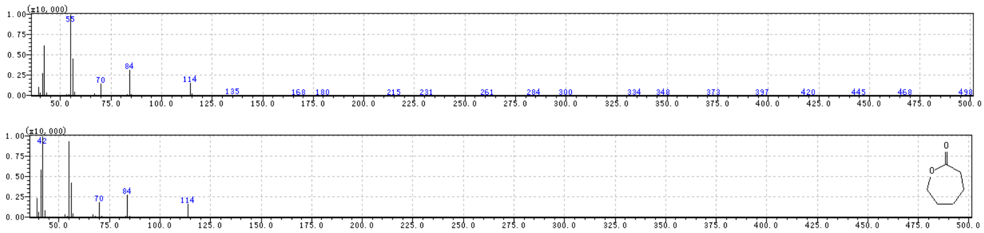

GC-MS: calcd. for $C_6H_{10}O_2$ 114, found 114 ($C_6H_{10}O_2$). Table 4.

Note: the upper MS image is experimental result, the lower is reference image from GC-MS library.

3) Entry 3, Table 4

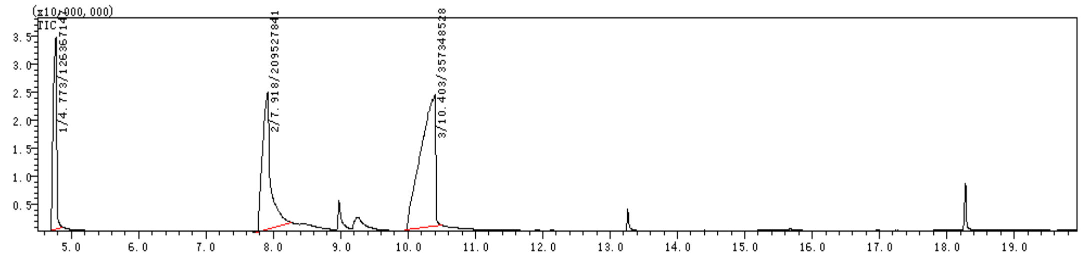

Figure S5. GC part of GC-MS for Entry 3, Table 4.

The peak for t_R = 4.773 min is unreacted substrate (cyclohexanone), the peak for t_R = 7.918 min is Product (oxepan-2-one), Table 4.

The peak for t_R = 10.403 min is by-product of *m*-CPBA (*m*-chlorobenzoic acid), Table 4:

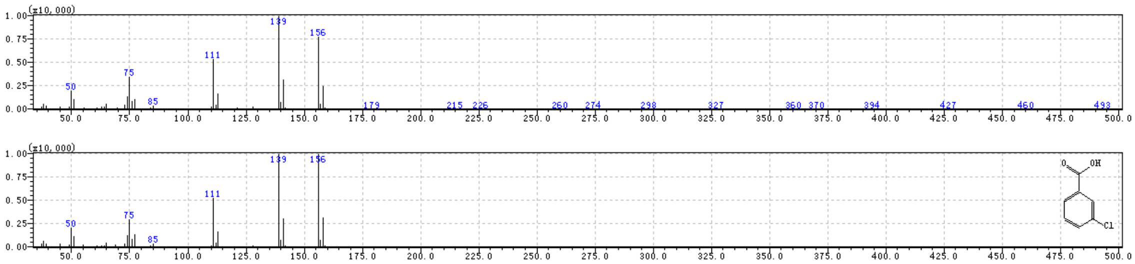

GC-MS: calcd. for $C_7H_5ClO_2$ 156, found 156 ($C_7H_5ClO_2$). Table 4.

4) Entry 4, Table 4

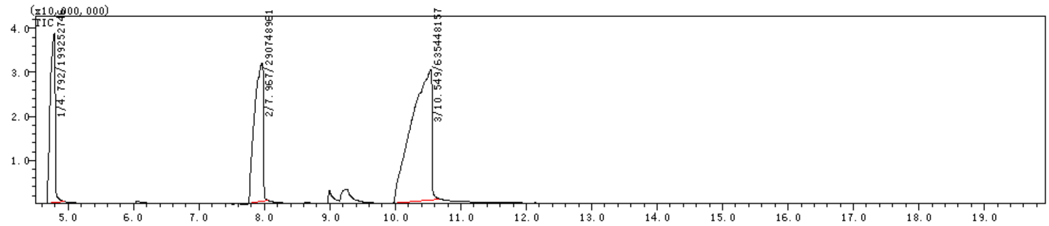

Figure S6. GC part of GC-MS for Entry 4, Table 4.

The peak for t_R = 4.792 min is unreacted substrate (cyclohexanone), the peak for t_R = 7.967 min is Product (oxepan-2-one), Table 4. The peak for t_R = 10.549 min is by-product of *m*-CPBA (*m*-chlorobenzoic acid), Table 4.

5) Entry 5, Table 4

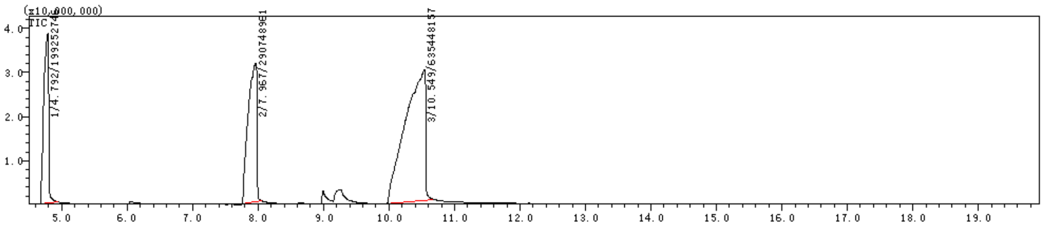

Figure S7. GC part of GC-MS for Entry 5, Table 4.

The peak for t_R = 4.792 min is unreacted substrate (cyclohexanone), the peak for t_R = 7.967 min is Product (oxepan-2-one), Table 4. The peak for t_R = 10.549 min is by-product of *m*-CPBA (*m*-chlorobenzoic acid), Table 4.

S4. GC-MS examples for Table 5

6) Entry 1, Table 5

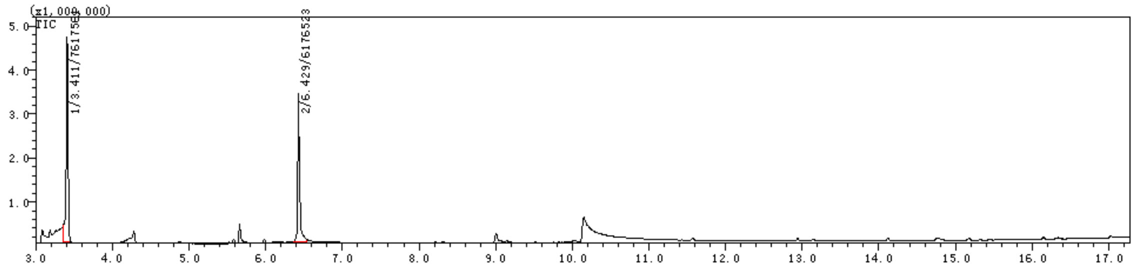

Figure S8. GC part of GC-MS for Entry 1, Table 5.

The peak for t_R = 3.411 min is unreacted substrate (cyclopentanone):

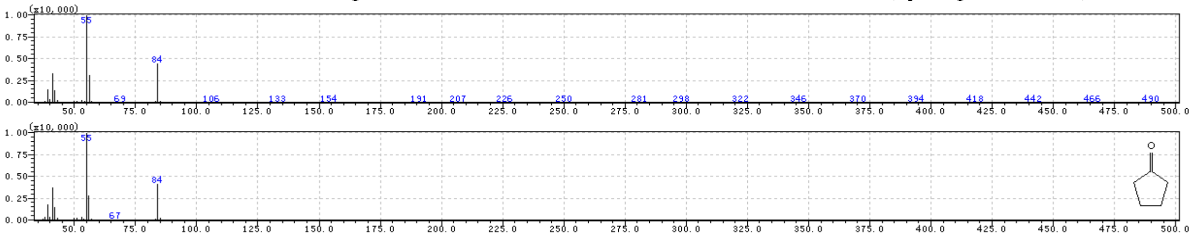

GC-MS: calcd. for C_5H_8O 84, found 84 (C_5H_8O). Table 5.

The peak for t_R = 6.429 min is Product (tetrahydropyran-2-one), Table 5:

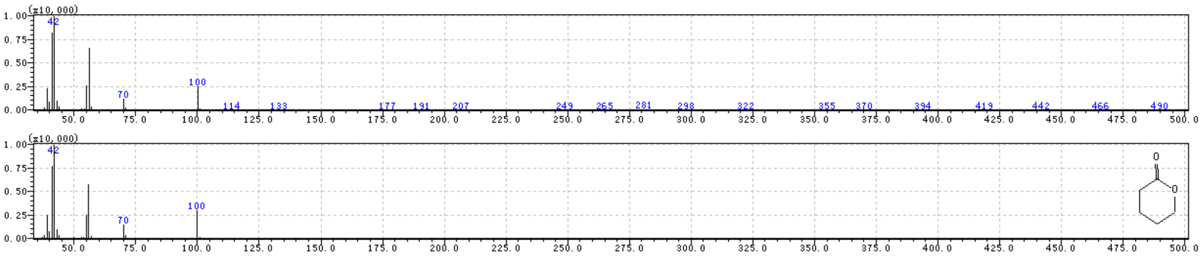

GC-MS: calcd. for $C_5H_8O_2$ 100, found 100 ($C_5H_8O_2$). Table 5.

The unmarked peak for t_R = 10.00-11.00 min is by-product of *m*-CPBA (*m*-chlorobenzoic acid).

7) Entry 3, Table 5

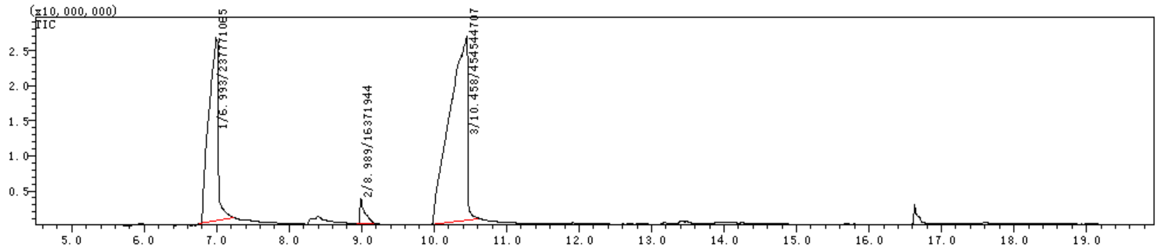

Figure S9. GC part of GC-MS for Entry 3, Table 5.

The peak for t_R = 6.993 min is Product (tetrahydropyran-2-one), Table 5, the peak for t_R = 10.458 min is by-product of *m*-CPBA (*m*-chlorobenzoic acid).

The peak for t_R = 8.989 min is an esterification product of *m*-chlorobenzoic acid:

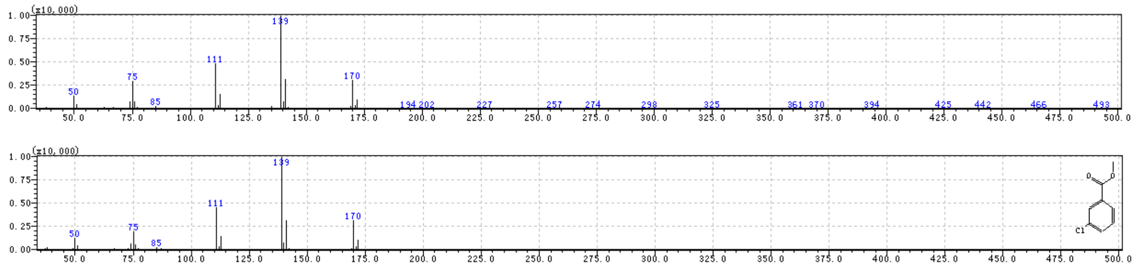

This by-product is derived from *m*-CPBA-induced oxidation, probably due to radical reaction with some impurities of solvent or reagents.

8) Entry 4, Table 5

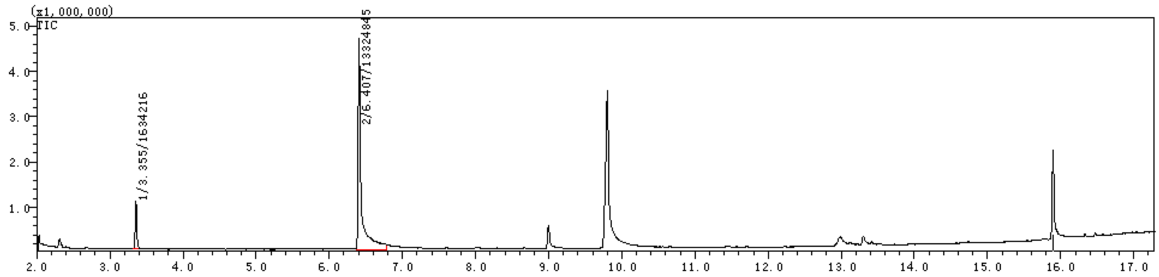

Figure S10. GC part of GC-MS for Entry 4, Table 5.

The peak for t_R = 3.355 min is unreacted substrate (cyclopentanone), the peak for t_R = 6.407 min is Product (tetrahydropyran-2-one), Table 5, the unmarked peak for t_R = 9.8-10.0 min is by-product of *m*-CPBA (*m*-chlorobenzoic acid).

S5. GC-MS examples for Table 6

9) Entry 2, Table 6

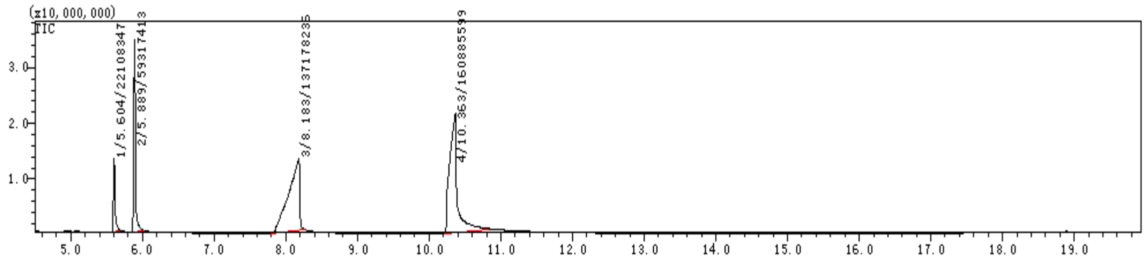

Figure S11. GC part of GC-MS for Entry 2, Table 6.

The peak for t_R = 5.604 min is unreacted substrate (cyclohexanecarbaldehyde):

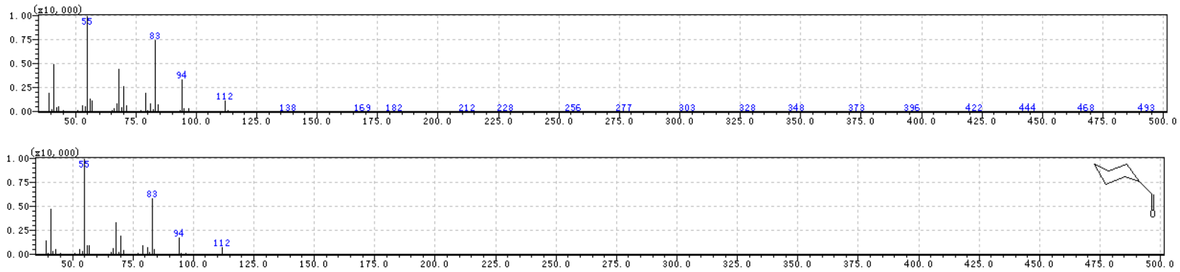

GC-MS: calcd. for $C_7H_{12}O$ 112, found 112 ($C_7H_{12}O$). Table 6.

The peak for t_R = 5.889 min is Product A (cyclohexylformate):

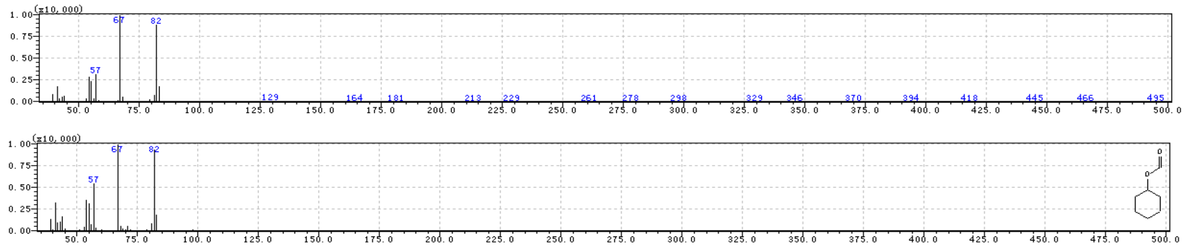

GC-MS: calcd. for $C_7H_{12}O_2$ 128, found 129 ($C_7H_{12}O_2$ + H). Table 6.

The peak for t_R = 8.183 min is Product B (cyclohexanecarboxylic acid), Table 6:

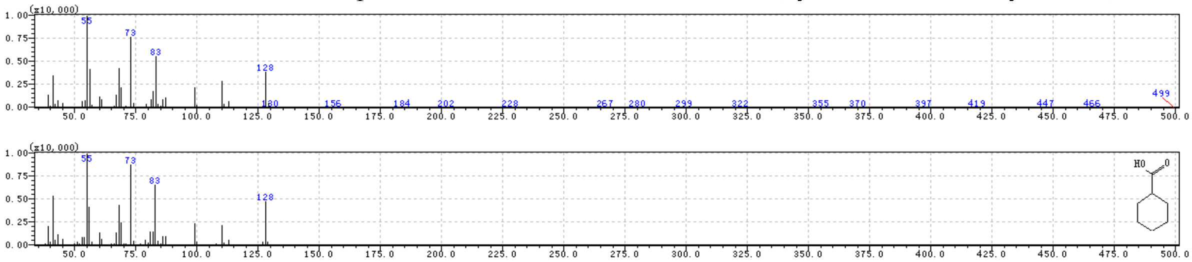

GC-MS: calcd. for $C_7H_{12}O_2$ 128, found 128 ($C_7H_{12}O_2$). Table 6.

The peak for t_R = 10.363 min is by-product of *m*-CPBA (*m*-chlorobenzoic acid), Table 6:

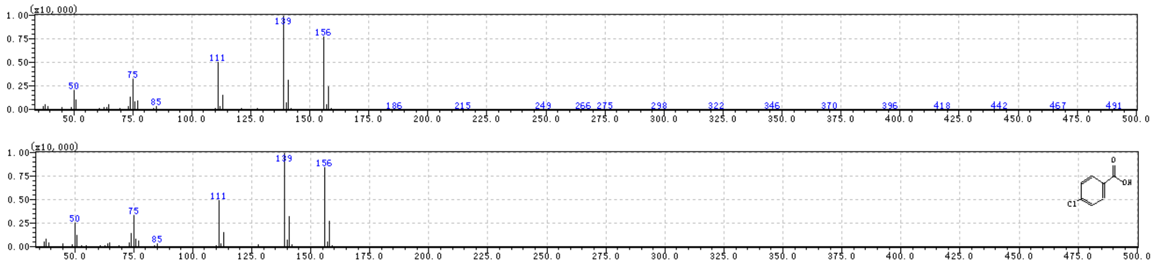

GC-MS: calcd. for $C_7H_5ClO_2$ 156, found 156 ($C_7H_5ClO_2$). Table 6.

10) Entry 4, Table 6

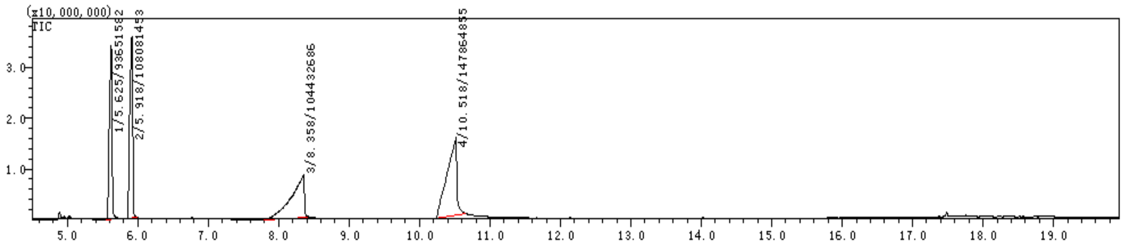

Figure S12. GC part of GC-MS for Entry 4, Table 6.

The peak for t_R = 5.625 min is unreacted substrate (cyclohexanecarbaldehyde), the peak for t_R = 5.918 min is Product A (cyclohexylformate), the peak for t_R = 8.358 min is Product B (cyclohexanecarboxylic acid), Table 6. The peak for t_R = 10.518 min is by-product of *m*-CPBA (*m*-chlorobenzoic acid), Table 6.

S6. GC-MS examples for Table 7

11) Entry 2, Table 7

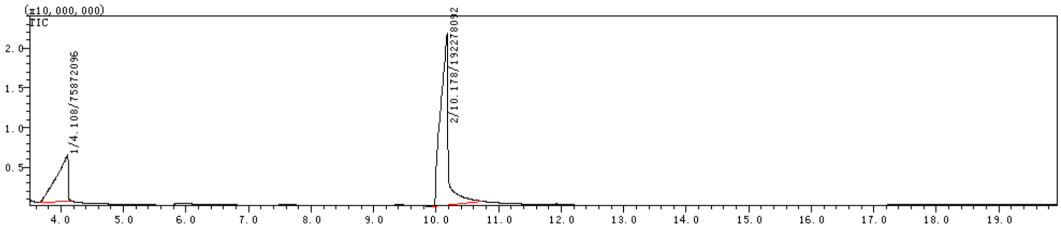

Figure S13. GC part of GC-MS for Entry 2, Table 7.

The peak for t_R = 4.108 min is *n*-butyric acid (Product, Table 7):

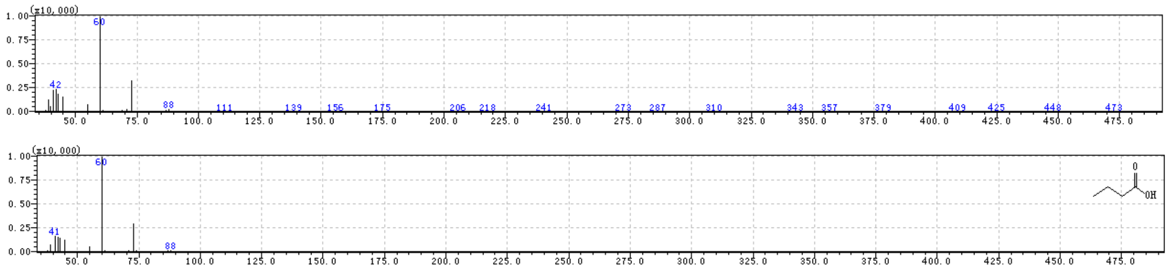

GC-MS: calcd. for $C_4H_8O_2$ 88, found 88 ($C_4H_8O_2$). Table 7.

The peak for t_R = 10.178 min is by-product of *m*-CPBA (*m*-chlorobenzoic acid), Table 7.

12) Entry 9, Table 7

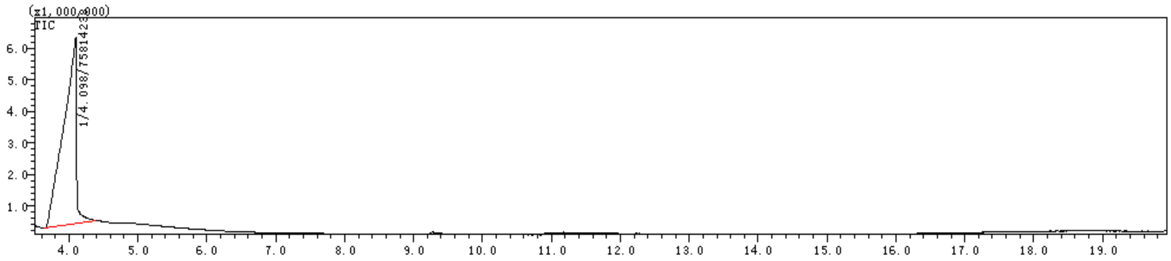

Figure S14. GC part of GC-MS for Entry 9, Table 7.

The peak for t_R = 4.098 min is *n*-butyric acid (Product, Table 7).

S7. GC-MS examples for Table 8

13) Entry 4, Table 8

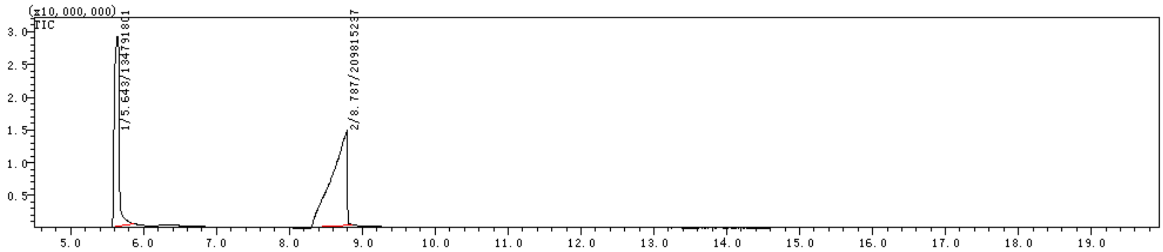

Figure S15. GC part of GC-MS for Entry 4, Table 8.

The peak for t_R = 5.643 min is benzaldehyde (unreacted substrate, Table 8):

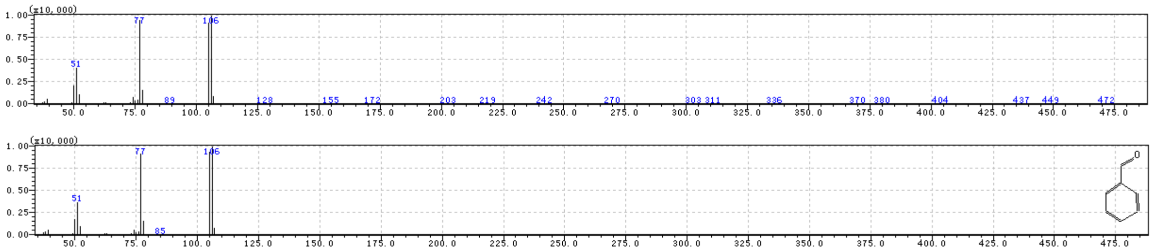

GC-MS: calcd. for C_7H_6O 106, found 106 (C_7H_6O). Table 8.

The peak for t_R = 8.787 min is benzoic acid (Product, Table 8):

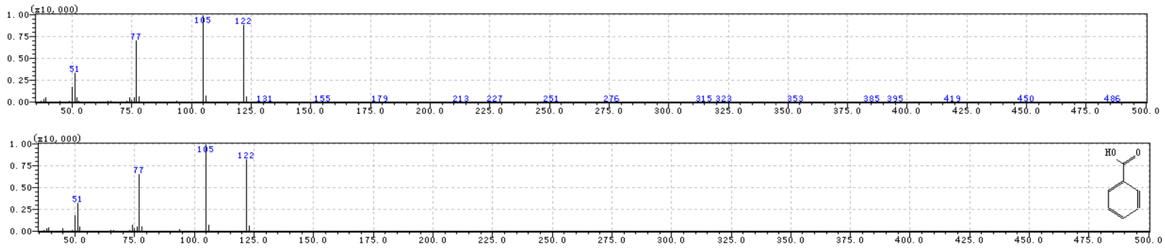

GC-MS: calcd. for $C_7H_6O_2$ 122, found 122 ($C_7H_6O_2$). Table 8.

14) Entry 5, Table 8

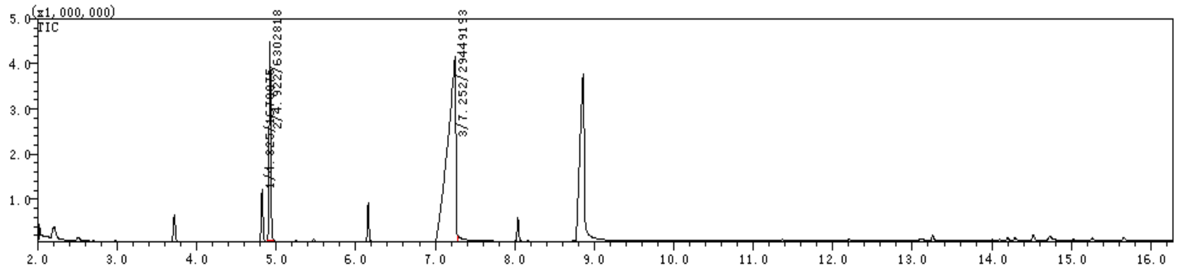

Figure S16. GC part of GC-MS for Entry 5, Table 8.

The peak for t_R = 4.886 min is benzaldehyde (unreacted substrate, Table 8):

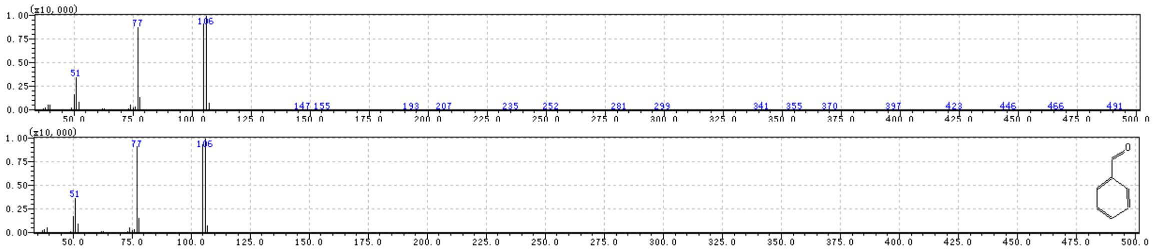

GC-MS: calcd. for C_7H_6O 106, found 106 (C_7H_6O). Table 8.

The peak for t_R = 4.922 min is phenethyl ether (by-product, Table 8):

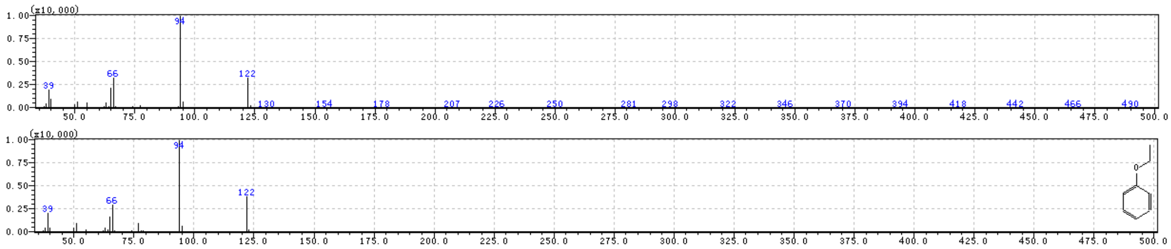

GC-MS: calcd. for $C_8H_{10}O$ 122, found 122 ($C_8H_{10}O$). Table 8.

The peak for t_R = 7.252 min is benzoic acid (Product, Table 8):

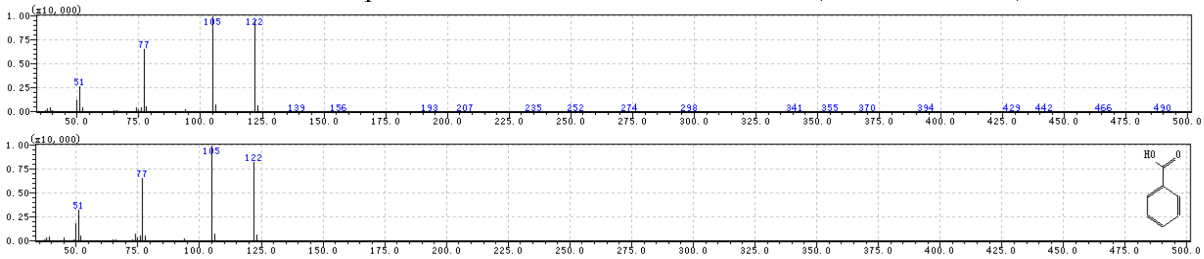

GC-MS: calcd. for $C_7H_6O_2$ 122, found 122 ($C_7H_6O_2$). Table 8.

S8. Optimized structures (distance in Å) for the oxidation of cyclopentanone under H_2O_2 catalyzed by C3 and Sn-beta zeolite model

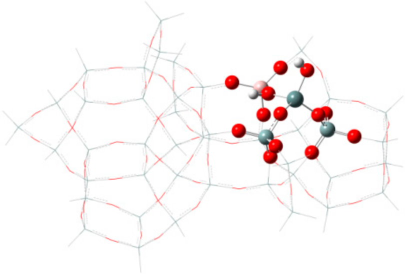

Al-Cat

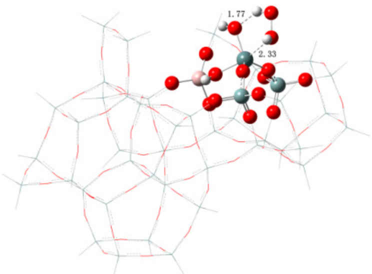

Al-Int-1

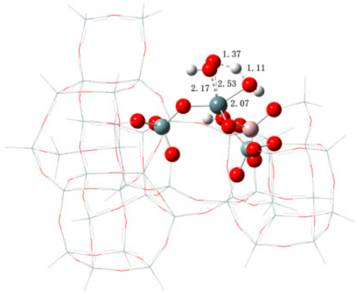

Al-TS-1

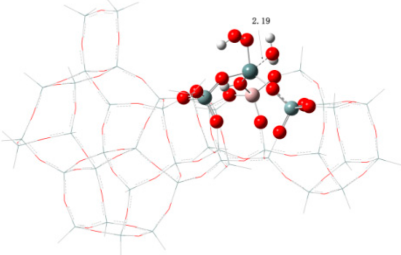

Al-Int-2

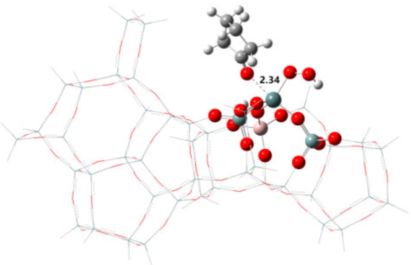

Al-Int-3

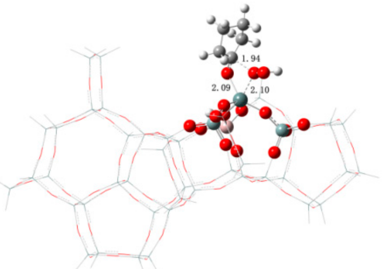

Al-TS-2

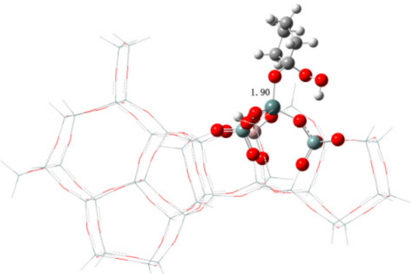

Al-Int-4

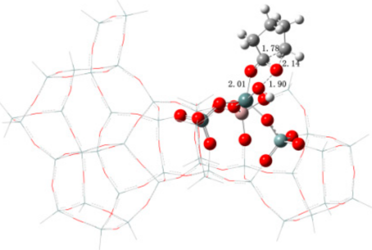

Al-TS-3

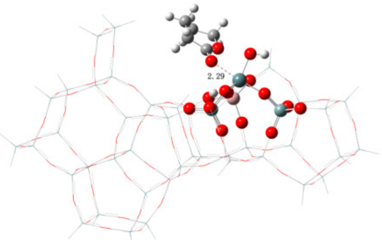

Al-Int-5

Figure S17. Optimized structures (distance in Å) for the oxidation of cyclopentanone with H_2O_2 catalyzed by C3.

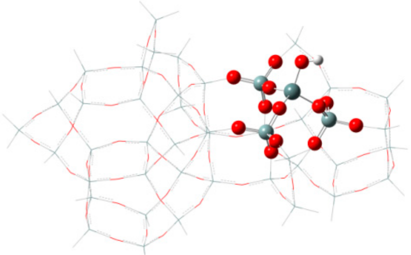

Si-Cat

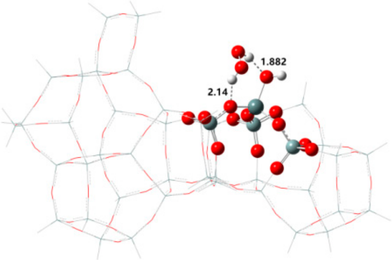

Si-Int-1

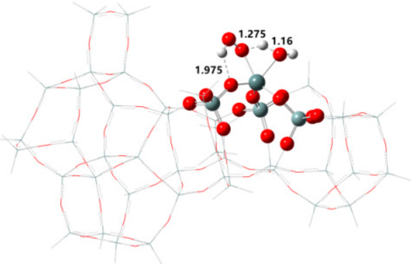

Si-TS-1

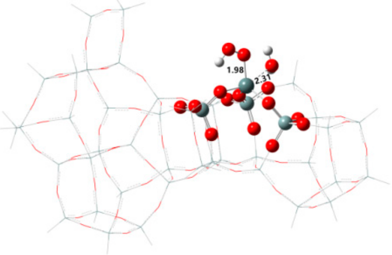

Si-Int-2

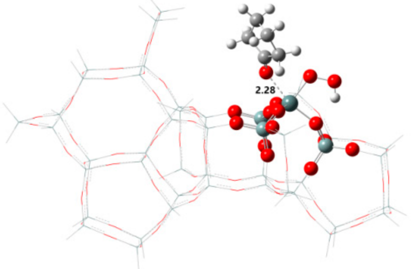

Si-Int-3

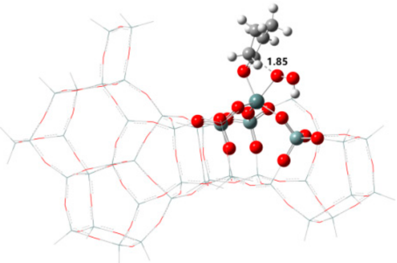

Si-TS-2

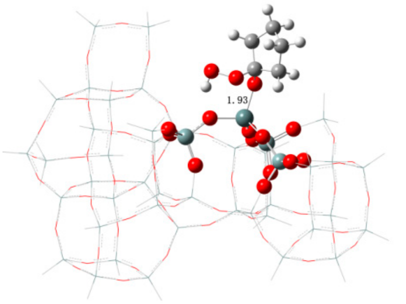

Si-Int-4

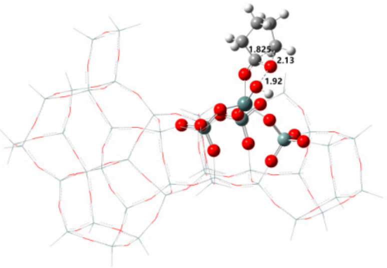

Si-TS-3

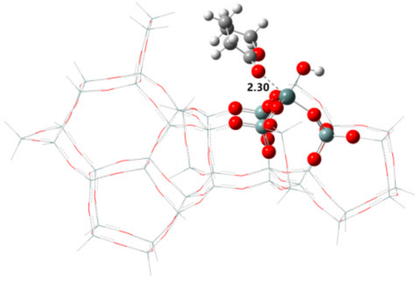

Si-Int-5

Figure S18. Optimized structures (distance in Å) for the oxidation of cyclopentanone with H_2O_2 catalyzed by Sn-beta zeolite model.
